# Cannabidiol in Skin Health: A Comprehensive Review of Topical Applications in Dermatology and Cosmetic Science

**DOI:** 10.3390/biom15091219

**Published:** 2025-08-23

**Authors:** Aura Rusu, Andreea-Maria Farcaș, Octavia-Laura Oancea, Corneliu Tanase

**Affiliations:** 1 Pharmaceutical and Therapeutic Chemistry Department, Faculty of Pharmacy, George Emil Palade University of Medicine, Pharmacy, Science and Technology of Târgu Mureș, 540142 Târgu Mureș, Romania; aura.rusu@umfst.ro (A.R.); andreeafarcas369@yahoo.com (A.-M.F.); 2 Organic Chemistry Department, Faculty of Pharmacy, George Emil Palade University of Medicine, Pharmacy, Science and Technology of Târgu Mureș, 540142 Târgu Mureș, Romania; 3 Pharmaceutical Botany Department, Faculty of Pharmacy, George Emil Palade University of Medicine, Pharmacy, Science and Technology of Târgu Mureș, 540142 Târgu Mureș, Romania; corneliu.tanase@umfst.ro

**Keywords:** cannabidiol, cannabinoids, phytocannabinoids, topical application, analgesic, anti-inflammatory, antioxidant, antibacterial, antiproliferative, skin rejuvenation, skin protection

## Abstract

Cannabidiol (CBD), a non-psychoactive phytocannabinoid derived from *Cannabis sativa* L., has emerged as a promising multifunctional agent in dermatology and cosmetic science. The review provides an updated synthesis of CBD’s topical therapeutic potential, challenges, and evolving regulatory frameworks. CBD exhibits diverse biological effects, including anti-inflammatory, antioxidant, antibacterial, analgesic, lipostatic, antiproliferative, moisturising, and anti-ageing properties through interactions with the skin’s endocannabinoid system (ECS), modulating CB1, CB2, TRPV channels, and PPARs. Preclinical and clinical evidence support its efficacy in managing acne, psoriasis (including scalp psoriasis), atopic and seborrheic dermatitis, and allergic contact dermatitis. CBD also relieves pruritus through neuroimmune modulation and promotes wound healing in conditions such as pyoderma gangrenosum and epidermolysis bullosa. In hair disorders such as androgenetic alopecia, it aids follicular regeneration. CBD shows promise in managing skin cancers (melanoma, squamous cell carcinoma, Kaposi sarcoma) and pigmentation disorders such as melasma and vitiligo. It enhances skin rejuvenation by reducing oxidative stress and boosting collagen and hydration. However, there are challenges regarding CBD’s physicochemical stability, skin penetration, and regulatory standardisation. As consumer demand for natural, multifunctional skincare grows, further research is essential to validate its long-term safety, efficacy, and optimal formulation strategies.

## 1. Introduction

### 1.1. General Aspects of the Use of Cannabis sp.

*Cannabis sativa* L. plant, a member of the Cannabaceae family, originates from Central and South Asia. *Cannabis sativa* L. is categorised into several subspecies, including *sativa, indica, kafiristanca, ruderalis,* and *spontanea* subsp. [1]. Because the boundaries between them are fluid and there is no universally recognised taxonomic rank, it has been suggested that only one species of cannabis, *Cannabis sativa*, should be recognised (cannabis) [2,3]. The *Cannabis* plant has been used to produce hemp fibres (for clothing, rope, and paper) and seeds, which can be used as animal feed. Still, it is also used for medicinal, recreational or religious practices [3,4].

In Western culture, several expressions have been adopted for this plant, depending on its use and composition. For example, “hemp” or “industrial hemp” are terms used to classify varieties of *Cannabis sativa* that contain 0.3% or less tetrahydrocannabinol (Δ^9^–THC), the primary psychoactive compound in the plant. In comparison, “marijuana” or “medical cannabis” can contain up to 30% Δ^9^–THC and is considered a prohibited substance [3].

At the end of the 19th century, cannabinol (CBN) became the first phytocannabinoid to be isolated, derived from a red oil extract of cannabis. Its structure was determined in the early 1930s, and by 1940, its chemical synthesis had been accomplished. The same year, (−)-cannabidiol (CBD) was also isolated, possibly along with cannabidiolic acid. In 1942, the extraction of Δ^9^–THC, likely a combination of (−)-Δ^8^– and (−)-Δ^9^– THC, was achieved, further advancing the understanding of cannabis compounds. In 1963, the structures and stereochemistry of cannabidiol (CBD) were clarified, followed by the elucidation of Δ^9^–Δ^9^–THC’s structure in 1964, marking its first isolation from cannabis. Further advancements came in 1965 with the synthesis of (±)-Δ^9^– THC and (±)-CBD, which paved the way for synthesising the (+)- and (−)-enantiomers of both these cannabinoids, as well as Δ^8^– THC [5]. Another pivotal advance came in 1988, with the discovery of the first cannabinoid receptor (CB1). These discoveries laid the foundation for modern cannabinoid research and marked a milestone, paving the way for deeper exploration into the effects and mechanisms of cannabis [1,5].

The *Cannabis sativa* plant contains 278 cannabinoid molecules and other phytochemical compounds (221 terpenoids, 174 terpenes, 92 steroids, 63 flavonoid glycosides, 46 polyphenols, and 19 flavonoids) contributing to its biological effects on the body [6]. The two major neuroactive components in cannabis plants are the primary psychoactive alkaloid, Δ^9^–THC, and the non-psychoactive alkaloid, CBD [4,7].

In recent years, there has been an increase in the development and use of more cannabis-derived products for therapeutic or cosmetic applications. Growing interest in cannabinoids or other bioactive compounds extracted from the cannabis plant has been driven by their potential therapeutic effects and their beneficial properties for skin health. Thus, cannabinoids are increasingly being incorporated into dermatological and cosmetic products [7,8].

Current in vitro and in vivo research indicates that CBD exhibits properties such as antioxidant, anti-inflammatory, moisturising, anti-acne, wound-healing, skin protection and anti-ageing effects [9,10]. In addition, CBD has demonstrated the ability to suppress bacterial growth, including that of *Cutibacterium acnes*, a key bacterium involved in the formation of acne. Its antibacterial action, coupled with its lipostatic, anti-inflammatory, and antiproliferative properties, highlights CBD’s potential as an effective agent in acne management [11,12]. In cosmetic formulations, full-spectrum and broad-spectrum hemp extracts are commonly used; these extracts predominantly contain CBD, along with minor cannabinoids such as CBG, CBC, and CBDV, depending on the extraction method and plant chemotype [12]. CBD is typically the primary active compound due to its well-established anti-inflammatory, antioxidant, and moisturising effects [9,12].

However, there is a limited number of clinical studies focused on its application in skincare. Additionally, the development of an effective drug-delivery system for CBD’s topical or transdermal use remains a pressing need. More comprehensive investigations, including clinical trials and pharmacokinetic analyses, are necessary to thoroughly understand CBD’s potential in cosmetic dermatology [9,10].

Due to its therapeutic potential, CBD is a promising candidate for treating various dermatological and inflammatory conditions (e.g., atopic dermatitis, psoriasis, acne, and wound healing). Topical administration allows CBD to act directly at the site of application, reducing systemic side effects and enhancing localised therapeutic outcomes. Thus, CBD faces challenges in permeating the skin barrier due to its lipophilic nature. However, advancements in formulation technologies (e.g., nanoencapsulation) are being explored to improve its bioavailability and efficacy. The synergistic effects of CBD and Δ^9^–THC have been noted to enhance therapeutic outcomes while minimising adverse effects, making this combination a viable option for specific conditions. CBD is generally well-tolerated, although some mild adverse effects, such as skin irritation, have been reported. Further studies are needed to establish standardised dosages and formulations [13].

### 1.2. Regulations of Cannabidiol

As a cannabinoid, CBD is subject to numerous regulations. In the European Union, CBD is classified as a “novel food” under Regulation (EC) No. 2015/2283. Thus, CBD products require prior risk assessment and authorisation before being placed on the market [1,14].

Products containing CBD must adhere to strict Δ^9^–THC limits. CBD products must contain less than 0.2% Δ^9^–THC to comply with European Union (EU) regulations. In the United Kingdom (UK), the threshold for Δ^9^–THC in products is set at 1 mg per container. This limit ensures that the products are non-psychoactive and safe for consumption. There is no unified legislation across the EU regarding CBD use. National regulatory entities often have differing stances, particularly on the decriminalisation and commercialisation of CBD products. CBD is found in various forms, including oils, capsules, sprays, and cosmetics. Each product category is subject to specific legal and quality requirements [1,14].

CBD products may be classified as medicines if they are marketed with claims of treating or preventing diseases or if their composition aligns with doses found in licensed drugs. The Human Medicines Regulation in the UK governs this classification. In the UK, CBD may fall under the Psychoactive Substances Act if it affects the central nervous system and mental functioning. However, foods containing CBD are exempt if they are considered “ordinarily consumed.” The UK Food Standards Agency (FSA) has set an upper intake limit of 70 mg/day for CBD, based on safety assessments. Products exceeding this limit may be deemed unsafe [14].

The regulation of CBD in the United States of America (USA) is complex and varies significantly between federal and state levels. Federally, the 2018 Farm Bill legalised the sale of hemp-derived products, including CBD, provided they contain less than 0.3% THC. However, most CBD products on the market are not Food and Drug Administration (FDA)-approved and lack rigorous testing for safety and efficacy. The DEA classifies all marijuana-derived products as Schedule I substances, but hemp-derived CBD is legal under federal law. State regulations differ widely, with some states allowing both medical and recreational use of cannabis products, while others only permit CBD with little to no THC for medical purposes. The lack of consistent regulation leads to variability in product quality and labelling, posing challenges for healthcare providers in advising patients on safe and legal CBD use. Healthcare professionals must stay informed about both federal and state laws to guide patients effectively [15].

The FDA does not allow CBD to be sold as a dietary supplement. Currently, the FDA has approved two CBD-based products: Sativex and Epidiolex [1,9,16]. Sativex oral spray, a cannabis plant extract containing equal proportions of CBD (2.5 mg/μL) and Δ^9^–THC (2.7 mg/μL), is approved for use in 17 EU member states and Norway. It is administered buccally or sublingually for the treatment of muscle spasticity associated with multiple sclerosis [1,15,17]. Epidiolex, containing CBD (100 mg/mL), received FDA approval in 2018 for managing seizures related to two severe epilepsy types: Lennox–Gastaut syndrome and Dravet syndrome. In 2020, its approval was extended to include the treatment of migraines caused by tuberous sclerosis complex, a rare genetic disorder that leads to the growth of benign brain tumours. Notably, CBD had not been utilised in food or dietary supplements before its incorporation as the active ingredient in Epidiolex [1,15,18,19].

Strict analytical procedures are essential to confirm that CBD products comply with safety standards, with particular attention to Δ^9^–THC levels. The process involves evaluating product purity and adherence to proper manufacturing practices. Natural CBD is permitted in cosmetics only when derived from cannabis extracts and tinctures originating from seeds and leaves, not associated with the fruiting tops of the plant. Harmonised standards across the EU are lacking, leading to varied interpretations and enforcement [1,14].

In the USA, topical CBD products, such as creams, balms, and lotions, are subject to the same federal regulations as other CBD products. Under the 2018 Farm Bill, these products are legal if they are derived from hemp and contain less than 0.3% THC by dry weight. However, the FDA has not approved most CBD products, including topicals, which means they are not subject to the same rigorous testing for safety and efficacy as FDA-approved drugs. State regulations for topical CBD products can vary widely. Some states have more lenient laws, allowing the sale and use of CBD topicals for both medical and recreational purposes, while others have stricter regulations that may limit their availability. Consumers need to check their local laws and ensure they purchase products from reputable sources that provide clear labelling and undergo third-party testing to verify CBD content and purity [15].

In Australia, cannabis-based registered medicines undergo a centralised approval process through the Therapeutic Goods Administration (TGA), which requires evidence of quality, safety, and efficacy before they are listed on the Australian Register of Therapeutic Goods (ARTG). However, most medicinal cannabis products are cannabis-based non-registered medicines, which are not assessed or listed on the ARTG but can still be legally accessed under specific conditions. The TGA facilitates access to cannabis-based non-registered medicines via dedicated pathways such as the Special Access Scheme (SAS) and the Authorised Prescriber Scheme, allowing doctors to prescribe unapproved cannabis products for serious or life-threatening conditions. By mid-2019, over 11,000 SAS applications had been approved for conditions like neuropathic pain and refractory paediatric epilepsy [19].

Efforts to reschedule cannabis preparations internationally, including CBD products with less than 0.2% Δ^9^–THC, could impact their regulatory status. However, resistance from the EU may delay such changes [14].

Regulatory agencies in the EU, USA, and Australia generally adopt similar approaches to cannabis-based medicinal products that are classified as medicines, treating them under the same framework as other regulated pharmaceuticals. However, key differences exist. In the EU, the regulatory structure involves a networked system with national authorities playing a significant role, particularly in overseeing Herbal Medicinal Products. In contrast, the USA and the EU differ in how they handle cannabis-based products that are not formally approved as medicines, with regulation occurring at the federal (FDA/EMA) or state/national level. Additionally, Australia provides specific access pathways for unapproved cannabis-based medicinal products through the TGA [19].

### 1.3. Aim of the Paper

This review aims to provide a comprehensive and up-to-date synthesis of the current scientific knowledge regarding the topical application of CBD in dermatology and cosmetic science. Thus, the paper explores the multifaceted biological effects of CBD on the skin through its interaction with the endocannabinoid system (ECS) and other molecular pathways. Based on biological effects, the paper aims to examine the therapeutic potential of CBD in managing various dermatological conditions, as well as its role in skin rejuvenation and protection. Furthermore, it discusses the challenges related to CBD’s physicochemical properties, delivery systems, regulatory frameworks, and safety profile, while highlighting future directions for research, innovation, and clinical application in the cosmetic and dermatological fields.

## 2. Materials and Methods

The review is structured as a narrative bibliographic review, designed to synthesise and critically interpret existing literature on the topical application of CBD in dermatology and cosmetic science. The literature reviewed in the manuscript was obtained from multiple scientific databases, including Clarivate Analytics, ScienceDirect, PubMed, and Google Books. Key search terms included “Cannabidiol”, “CBD”, “Cannabinoids”, “Phytocannabinoids”, and “Endocannabinoid system”, combined with dermatology- and cosmetic-related terms. Additional keywords such as “Topical application”, “Skin health”, “Analgesic”, “Antibacterial”, “Anti-inflammatory”, “Antioxidant”, “Antiproliferative”, “Lipostatic”, “Melanogenesis”, “Moisturising”, and “Skin protection” were also utilised. From the gathered literature, studies specifically addressing the topical use of cannabidiol were selected. Chemical structures were illustrated using Biovia Draw 2024 (https://discover.3ds.com/biovia-draw-academic-thank-you (accessed on 28 February 2025)) [20], while specific parameters were verified with MarvinSketch 23.10 (https://chemaxon.com/marvin (accessed on 28 February 2025)) [21]. IUPAC names for compounds were retrieved from the PubChem database (https://pubchem.ncbi.nlm.nih.gov/(accessed on 20 March 2025)) [22]. Grammarly Premium, version 6.8.261, was used exclusively for language editing purposes (https://www.grammarly.com/) [23].

## 3. Diverse Cannabinoids and Their Physicochemical Properties

### 3.1. General Chemical Aspects

The *Cannabis sativa* plant contains 278 cannabinoid compounds, among which are 150 phytocannabinoids [6]. Although numerous cannabinoid molecules have been identified in *Cannabis sativa* [6], detailed quantitative data on the percentage composition of individual CBD derivatives remain limited. Most studies focus on major cannabinoids such as CBD, Δ^9^–THC, CBG, CBC, and CBN, with their relative abundance assessed using techniques like High-Performance Liquid Chromatography (HPLC) and Gas Chromatography-Mass Spectrometry (GC-MS) [6,24].

There are three main classes of cannabinoids, namely:

- Phytocannabinoids, produced exclusively from *Cannabis sativa*;
- Endocannabinoids, which exist or are produced naturally in the human body;
- Synthetic cannabinoids, structurally similar to phytocannabinoids or endocannabinoids but synthesised in the laboratory [25].

Most phytocannabinoids have little or no psychoactive activity, and a significant portion have very few side effects, making them exciting candidates for the treatment of various diseases [26]. Three general categories of cannabinoids, based on their natural occurrence, are presented in Table 1; each category contains several subcategories based on molecular structure [27].

Cannabinoids can also be classified according to the presence of a carboxyl group, differentiating between neutral cannabinoids (such as Δ^9^–THC and CBD) and acidic cannabinoids. In plants, the concentrations of neutral cannabinoids are much lower compared to those of acidic cannabinoids. Thus, Δ^9^–THC and CBD are formed by non-enzymatic decarboxylation, resulting from stress events such as exposure to light, heating or ageing of their acidic precursors, Δ^9^-tetrahydrocannabinolic acid (THCA) and cannabidiolic acid (CBDA). The amount of each compound formed in the plant depends on genetic characteristics and environmental conditions such as temperature, humidity and soil nutrition. THCA synthase and CBDA synthase were the first cannabinoid synthases studied and represent potential targets for various biotechnological applications, given that they produce the direct precursors of pharmacologically active cannabinoids [28].

#### 3.1.1. Phytocannabinoids

Phytocannabinoids are naturally occurring chemical compounds produced exclusively in *Cannabis sativa* plants and their related species. Most of them have little or no psychoactive activity and, for the most part, have few and not very severe side effects. Phytocannabinoids are a group of agents analogous to terpenophenolic compounds, with lipophilic properties, found mainly in the resin secreted by the trichomes of female plants [3,27].

**Figure 1:**
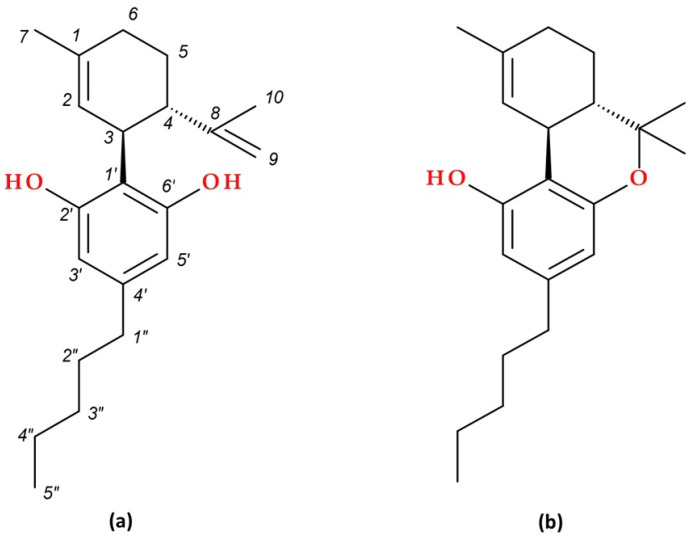
Molecular structures of: (**a**) (-)-Cannabidiol (CBD) and (**b**) (–)-*trans*-Δ9–Tetrahydrocannabinol (Δ^9^–THC) [22,24].

Phytocannabinoids are widely recognised for their significance, diverse health-related benefits, and psychoactive properties [6,29]; this highly heterogeneous group of pharmacologically active compounds is structurally and biochemically similar to the primary psychoactive compound derived from *Cannabis sativa*, Δ^9^–THC. The best-known representatives are CBD and Δ^9^–THC; their molecular structures are presented in Figure 1 [24,28,30].

CBD is known for its strong antagonistic effects on CB1 and CB2 receptor agonists. Unlike THC, which acts as a partial agonist at both CB1 and CB2 receptors with varying efficacy, CBD blocks these receptors and has been reported to act as an inverse agonist at both CB1 and CB2 receptors. Thus, CBD can potentially counteract the effects of other compounds that activate these receptors [31].

**Figure 2:**
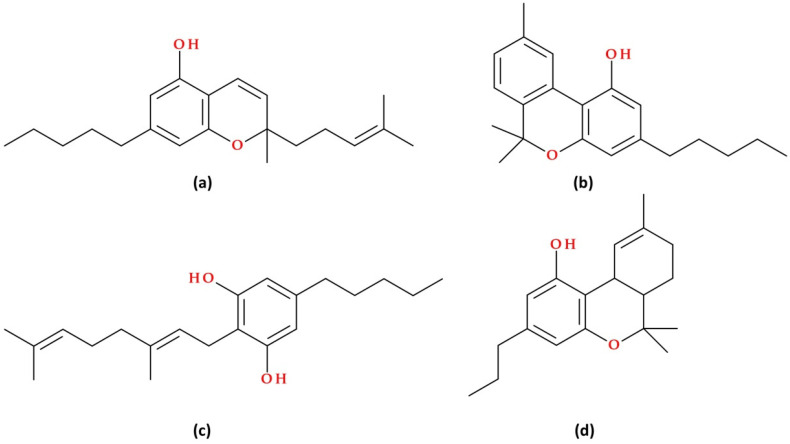
Molecular structures of other phytocannabinoids: (**a**) cannabichromene (CBC), (**b**) cannabinol (CBN), (**c**) cannabigerol (CBG), and (**d**) tetrahydrocannabivarin (THCV) [8,22,32,34].

The molecular structures of other phytocannabinoids are presented in Figure 2. Among them, phytocannabinoids with no psychotropic effects are cannabichromene (CBC) and cannabigerol (CBG). When ∆^9^– THC ages or is exposed to light, it undergoes oxidative breakdown, producing cannabinol (CBN); its psychotropic effects are mild [32]. Tetrahydrocannabivarin (THCV) has attracted particular attention due to its specific characteristics and non-psychoactive actions, which distinguish it from THC, its psychoactive analogue [33].

#### 3.1.2. Endocannabinoids

Endocannabinoids are natural chemical compounds produced by living organisms associated with cannabinoid receptors (CB1 and CB2) or generally considered part of the ECS. These are commonly referred to as neuromodulatory agents and have particular characteristics that distinguish them from typical neurotransmitters: they are synthesised at will at their site of action by receptor-stimulated cleavage of lipid membrane precursors. They are not conserved in synaptic vesicles [3,27]. Endocannabinoids are lipid mediators derived from membrane lipids in response to various signals, including inflammation triggers [35,36]. Endocannabinoids, which contain amides, esters, and ethers of long-chain polyunsaturated fatty acids, are lipid mediators isolated from the brain and peripheral organs [37].

**Figure 3:**
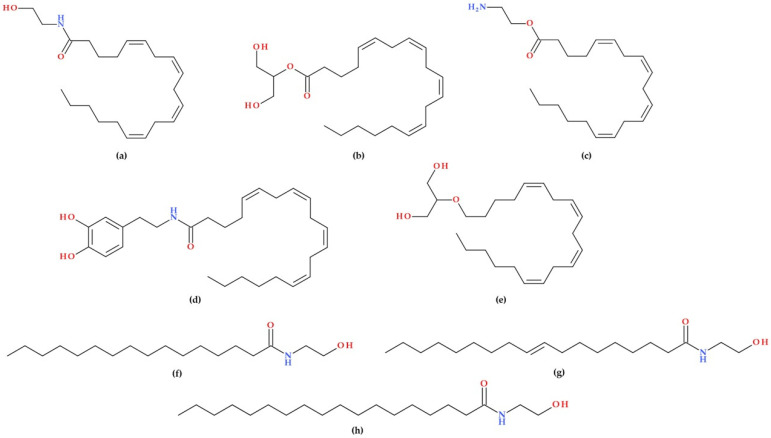
Endocannabinoids molecular structures: (**a**) anandamide (AEA), (**b**) 2-Arachidonoylglycerol (2-AG), (**c**) virodhamine, (**d**) N-arachidonoyl dopamine, (**e**) 2-Arachidonoyl glyceryl ether (noladin ether); “Endocannabinoid-like” molecular structures: (**f**) N-palmitoylethanolamine (PEA), (**g**) N-oleoylethanolamine (OEA), and (**h**) N-stearoylethanolamine [8,22].

Two primary endocannabinoids are anandamide (AEA) and 2-arachidonoylglycerol (2-AG). Δ^9^–THC and AEA share important pharmacophores [36,37]. AEA is a lipid neurotransmitter named after the Sanskrit word for “bliss”; it influences CB1 and CB2 receptors, promoting motivation and pleasure [35]. AEA acts as a partial agonist at CB1 and as a weak partial agonist at CB2 [31]. 2-AG, which binds more strongly to these receptors as a full agonist, was first identified in canine intestines [35]. Additionally, 2-AG has been identified as the primary endogenous ligand for CB2 [31]. Other endocannabinoid-like molecules, such as virodhamine and N-arachidonoyl dopamine, have been discovered, but their exact roles remain unclear [35]. Another endocannabinoid, 2-arachidonoyl glyceryl ether (noladin ether), has been demonstrated to be a full agonist at CB2 [31,38]. The “endocannabinoid-like” substances N-palmitoylethanolamine (PEA), N-oleoylethanolamine (OEA), and N-stearoylethanolamine are found in significant quantities in the brains of humans, mice, and rats [37]. The molecular structures of the most well-known endocannabinoids and “endocannabinoid-like” molecules are shown in Figure 3.

#### 3.1.3. Synthetic Cannabinoids

Intensive research into phytocannabinoids and endocannabinoids has led to the development of synthetic compounds with high potency and selectivity for cannabinoid receptors. These synthetic cannabinoids offer new opportunities for targeting the ECS with selective receptor ligands that have enhanced pharmacotherapeutic potential and limited toxicity [31]. Unlike other types of cannabinoids, synthetic ones are chemical compounds developed in laboratories which have structural similarities to both endocannabinoids and phytocannabinoids and act through similar biological mechanisms [3].

**Figure 4:**
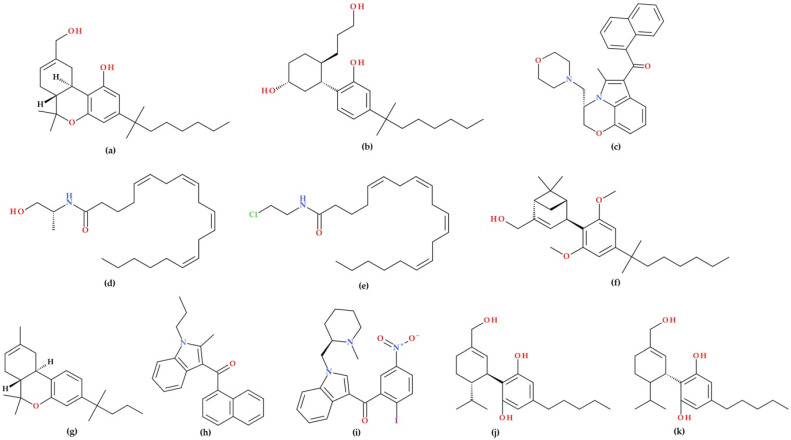
Synthetic cannabinoids molecular structures: (**a**) HU-210 (IUPAC name: (6*aR*,10*aR*)-9-(hydroxymethyl)-6,6-dimethyl-3-(2-methyloctan-2-yl)-6*a*,7,10,10*a*-tetrahydrobenzo[c]chromen-1-ol), (**b**) CP-55940 (IUPAC name: 2-[(1*R*,2*R*,5*R*)-5-hydroxy-2-(3-hydroxypropyl)cyclohexyl]-5-(2-methyloctan-2-yl)phenol), (**c**) R-(+)-WIN-55,212–2 (IUPAC name: [(11*R*)-2-methyl-11-(morpholin-4-ylmethyl)-9-oxa-1-azatricyclo [6.3.1.04,12]dodeca-2,4(12),5,7-tetraen-3-yl]-naphthalen-1-ylmethanone), (**d**) R(+)-methanandamide (IUPAC name: (5*Z*,8*Z*,11*Z*,14*Z*)-*N*-[(2*R*)-1-hydroxypropan-2-yl]icosa-5,8,11,14-tetraenamide), (**e**) ACEA (IUPAC name: (5*Z*,8*Z*,11*Z*,14*Z*)-*N*-(2-chloroethyl)icosa-5,8,11,14-tetraenamide); (**f**) HU-308 (IUPAC name: ([(1*S*,4*S*,5*S*)-4-[2,6-dimethoxy-4-(2-methyloctan-2-yl)phenyl]-6,6-dimethyl-2-bicyclo [3.1.1]hept-2-enyl]methanol), (**g**) JWH-133 (IUPAC name: (6*aR*,10*aR*)-6,6,9-trimethyl-3-(2-methylpentan-2-yl)-6*a*,7,10,10*a*-tetrahydrobenzo[c]chromene), (**h**) JWH-015 (IUPAC name: (2-methyl-1-propylindol-3-yl)-naphthalen-1-ylmethanone), (**i**) AM1241 (IUPAC name: (2-iodo-5-nitrophenyl)-[1-[[(2*R*)-1-methylpiperidin-2-yl]methyl]indol-3-yl]methanone, (**j**) HU-446 (IUPAC name: 2-[(1*S*,6*S*)-3-(hydroxymethyl)-6-isopropyl-cyclohex-2-en-1-yl]-5-pentyl-benzene-1,3-diol), and (**k**) HU-465 (IUPAC name: 2-[(1*R*)-3-(hydroxymethyl)-6-isopropyl-cyclohex-2-en-1-yl]-5-pentyl-benzene-1,3-diol) [20,22,24,31].

These compounds include agonists and antagonists that bind directly to cannabinoid receptors, as well as agents that enhance the synthesis, reduce the reuptake, or inhibit the degradation of endocannabinoids. Cannabinoid receptor agonists are classified into classical cannabinoids, non-classical cannabinoids, aminoalkylindoles, and eicosanoids, each with distinct chemical structures and properties. Notable examples include HU-210, CP-55940, R-(+)-WIN-55,212–2, and synthetic CB1-selective agonists like R(+)-methanandamide and ACEA, as well as selective CB2 agonists such as HU-308, JWH-133, JWH-015, and AM1241 (Figure 4) [31].

In their review article, Morales P. et al. (2017) [24] focus on the therapeutic potential and synthetic development of cannabidiol (CBD) derivatives. CBD has shown potential in treating various diseases, prompting drug discovery programs to develop synthetic derivatives with improved properties, such as increased potency and efficacy. Derivatives were created targeting CBD metabolites (e.g., 7-COOH-CBD, 7-OH-CBD) and modified CBD’s chemical structure at specific sites (lipophilic chain, hydroxyl groups, and C7-methyl). Both (−)-CBD and (+)-CBD enantiomers have been synthesised for study. Unlike natural (−)-CBD analogues that lack affinity for CB1 and CB2 cannabinoid receptors, many (+)-CBD derivatives bind to these receptors, especially CB1. Hydrogenated forms, such as H2-CBD and H4-CBD, exhibit anti-inflammatory effects and affinity for the CB1 receptor, which differs from that of natural CBD. The dihydro-7-hydroxy-CBD enantiomers (HU-446 and HU-465, Figure 4) have demonstrated anti-inflammatory potential, with HU-465 exhibiting a unique binding affinity to both CB1 and CB2 receptors. These synthetic derivatives enhance CBD’s therapeutic utility, particularly in inflammatory and autoimmune conditions [24].

### 3.2. Chemistry and Physicochemical Properties of Cannabidiol (CBD)

CBD was first isolated from marijuana in 1940 by Roger Adams and his team in the United States [39] and from Indian hemp resin by Alexander Todd (Nobel laureate) and his team in the United Kingdom [40]. Initially, they proposed a partial structure based on chemical degradation and its relationship with cannabinol. The complete structure of CBD was accurately identified in 1963 [41] and in 1964, based on nuclear magnetic resonance (NMR) data. The absolute stereochemistry was confirmed in 1967 [40,42,43].

CBD (2-[(1*R*,6*R*)-3-methyl-6-prop-1-en-2-ylcyclohex-2-en-1-yl]-5-pentylbenzene-1,3-diol) is the main non-psychoactive ingredient in *Cannabis sativa*. It has a tricyclic structure, unlike Δ^9^–THC, and has two linked rings: a pyran ring (a six-membered ring with oxygen) and a phenol ring (a six-membered ring with an attached hydroxyl group). It comes in various stereoisomeric forms; the naturally occurring form is typically *trans*-CBD [44]. According to formal definitions, CBD has a tetrahydrobiphenyl skeleton, a bicyclic core that depicts an adduct consisting of the alkylresorcinol derivative olivetol and the monoterpene *p*-cymene. Through an acid-catalysed process, CBD can be transformed into Δ^9^– THC, a tricyclic dibenzopyran [45].

Mechoulam R. and Shvo Y. (2002) reported the IR spectra and NMR spectroscopy of CBD [41], and Jones P.J. et al. (1977) identified the crystalline structure of CBD [46,47]. It was discovered that the terpene and aromatic rings were nearly perpendicular to one another. In contrast, the two rings in the structure of Δ^9^–THC are nearly in the same plane. However, since the two rings in CBD rotate freely in solution and the gaseous state, the various conformations in the crystalline state might not influence the activity differences between CBD and Δ^9^–THC [43].

CBD is a lipophilic substance poorly soluble in water [6,48]. Its low aqueous solubility poses a severe formulation challenge [49,50]. The results obtained by Franco C. et al. (2022) highlight that CBD is unstable in realistic pharmaceutical and commercial formulations, especially when acidic co-formulants or additives are present, as these can promote the formation of by-products and alter its degradation pathways [51]. Bini A. et al. (2024) investigated how different decarboxylated cannabis chemotypes, including CBD, respond to sunlight exposure, focusing on photodegradation and optimal storage conditions. They found that while all cannabinoids degrade significantly under light, CBD is more resistant to light-induced breakdown compared to Δ^9^– THC and CBN. However, the presence of oxygen greatly accelerates degradation through oxidation, making inert atmosphere storage essential for preserving cannabinoid stability. Notably, the research confirmed that CBD does not convert into psychoactive Δ^9^– THC under light exposure, dispelling concerns about accidental THC contamination in CBD products [52].

The physicochemical properties of CBD are summarised in Table 2.

## 4. The Endocannabinoid System of the Skin

Recent studies have identified a unique ECS in the skin, characterised by endogenous ligands that bind to the CB1 and CB2 receptors. CB1 and CB2 are cannabinoid receptors that belong to the class of G protein-coupled receptors. The activities of both CB1 and CB2 receptors have been identified in dermal nerve cells, keratinocytes, hair follicle epithelial cells, and sebocytes [57].

The epidermal ECS maintains skin homeostasis and barrier integrity, regulating various neuro-immunoendocrine functions. Other skin receptors targeted by cannabinoids, such as transient receptor potential (TRP) channels and peroxisome proliferator-activated receptors (PPARs), exist in multiple skin cells. For instance, the transient receptor potential vanilloid receptor TRPV-4 is in eccrine sweat glands, TRPV-1 in keratinocytes, and TRPV-3 in hair follicles. PPAR-α is highly expressed in basal keratinocytes and hair papilla cells, while PPAR-δ and PPAR-γ are found in the epidermis, hair follicles, and sebaceous glands [12].

The presence of enzymes for synthesising and degrading endocannabinoids in the skin indicates that the cutaneous system is actively involved in cannabinoid metabolism. Sebocytes, melanocytes, fibroblasts, and immunocytes contain enzymes like fatty acid amide hydrolase and monoacylglycerol lipase. Examples of naturally occurring endocannabinoids are 2-AG, AEA, and PEA. The endocannabinoids 2-AG and AEA are abundant in the skin and have been extensively studied for their importance in skin-related research [12,57].

An imbalance in endocannabinoid homeostasis could play a role in skin disorders, including compromised skin barrier function seen in atopic dermatitis. The role of endocannabinoid signalling in skin cell growth, regulation, proliferation, apoptosis, and communication has been established, highlighting how disruptions in ECS homeostasis can lead to dermatologic conditions like asteatotic eczema, atopic dermatitis, irritant and allergic contact dermatitis, and chronic pruritus. The cutaneous ECS interacts harmoniously with the immune, neurological, and endocrine systems, which are crucial in maintaining skin health and functionality. Potential treatments for these skin disorders must be explored through modulation of the ECS [57].

## 5. Biological Effects of Cannabidiol on the Skin

CBD influences the body through its interaction with the ECS. Preclinical and clinical studies have contributed to understanding the therapeutic potential of CBD for numerous conditions [58,59].

**Figure 5:**
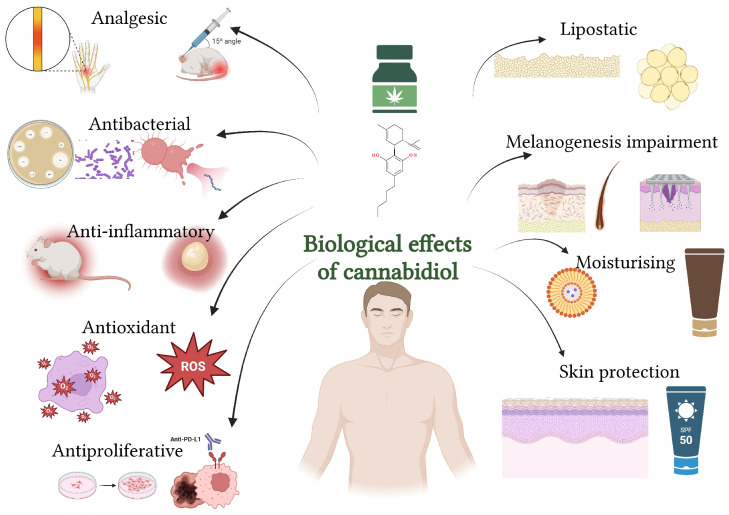
Biological effects of cannabidiol (CBD) on the skin (created with BioRender.com).

CBD exhibits several biological effects on the skin, including anti-inflammatory, antioxidant, antimicrobial, lipostatic, and wound-healing properties (Figure 5). It also shows potential in managing conditions like acne, eczema, psoriasis, and other dermatological disorders by modulating skin cell growth, reducing inflammation, and supporting skin barrier function (Section 7) [9,60,61].

The precise mechanisms of CBD’s actions are still not fully understood. CBD primarily interacts with the ECS through various mechanisms. Unlike endocannabinoids, CBD binds to the allosteric site of CB1 and CB2 receptors (low affinity), possibly serving as a negative allosteric modulator. It can also act as an antagonist or inverse agonist at these receptors and potentially as a partial agonist at CB2. CBD inhibits fatty acid amide hydrolase (FAAH), thereby increasing extracellular AEA levels, which activate the CB1 and CB2 receptors, as well as the TRPV-1 channel. It acts as an agonist for TRP channels, including TRPV1–4 and transient receptor potential ankyrin 1 (TRPA-1), as well as for the PPAR-γ. Also, CBD is an antagonist of GPR55 and TRPM8 [9,25,62]. Additionally, CBD acts as a partial agonist of the 5-HT1a receptor at low concentrations, transitioning to an inverse agonist at higher concentrations, and serves as an allosteric modulator for both μ and δ opioid receptors [62].

Topical applications of Δ^9^– THC and CBD have been shown to reduce pro-inflammatory cytokines (e.g., IL-6 and IL-17). Additionally, CBD pretreatment increases IL-10 levels, a cytokine that has anti-inflammatory properties. These immune-regulating effects seem to occur independently of cannabinoid receptor signalling pathways [62]. Many CBD products are used topically (balms, creams, gels, lotions, oils, patches, and salves). However, the capacity to assess the behavioural effects and perceived justification for using these products is limited by the absence of controlled clinical research and patient surveys [15].

### 5.1. Analgesic Effect

Endocannabinoids and synthetic cannabinoid agonists possess antinociceptive and anti-inflammatory properties. Although their effectiveness and safety are yet unknown, the use of cannabinoids to treat pain is growing [63]. Research, primarily conducted on lab animals, has demonstrated how CBD influences the perception of pain. CBD may have more potent effects than Δ^9^–THC, even though both cannabinoids have analgesic qualities [64].

CBD acts as an analgesic through multiple mechanisms. It interacts with various receptors, including CB1 and CB2, serotonin receptors (5HT1a), and TRP channels, modulating pain perception and inflammation. CBD’s weak binding to cannabinoid receptors suggests its analgesic effects are largely independent of these receptors, instead involving negative allosteric modulation. Additionally, CBD influences ion channels, such as TRPV-1 and TRPA-1, which play roles in pain signalling. It also inhibits the uptake of AEA, enhancing its pain-relieving effects. Furthermore, CBD’s anti-inflammatory properties contribute to its analgesic action by reducing pro-inflammatory cytokines and oxidative stress. These combined mechanisms make CBD a promising option for managing various types of pain, including neuropathic and inflammatory pain [64].

Maida V. and Corban J. (2017) evaluate topical medical cannabis as a new treatment for wound pain in three cases of pyoderma gangrenosum. The study found that applying topical medical cannabis resulted in significant pain relief and reduced the need for opioid analgesics in all three patients. For each patient, a different concentration of the combination CBD + Δ^9^–THC was used in the topical compound (Δ^9^–THC 5 mg/mL + CBD 6 mg/mL for one patient, and Δ^9^–THC 7 mg/mL + CBD 9 mg/mL for two patients). The authors suggest that topical medical cannabis has the potential to improve pain management in patients with various types of wounds, highlighting its rapid onset of analgesia and opioid-sparing effects. The study emphasises the need for further investigation through more extensive controlled trials to evaluate its efficacy and safety more comprehensively [65].

Hall N. et al. (2023) conducted a study that supported the development of topical CBD products, with participants reporting significant pain relief and decreased disability related to pain. Data from 20 individuals who had all played professional basketball, track and field, or American football for four to ten years were subjected to a retrospective analysis. To treat chronic pain brought on by recent lower extremity injuries, participants received topical CBD (10 mg twice daily via a controlled dispenser). During the 6-week trial period, self-reports of pain, pain-related impairment, activities of daily living, and tolerability assessments and secondary analyses of pain were gathered. This group responded favourably to topical CBD treatment, with just mild side effects. It will likely identify safety or tolerability issues, as professional athletes are trained to evaluate safety concerns. Significant pain reductions and functional gains were observed after six weeks of treatment. The information gathered from this pilot study supports the need for more prospective, randomised, and controlled research on topical CBD in professional athletes [66].

Pastina J.T. et al. (2024) explored the effects of cannabidiol (CBD) cream on muscle soreness and performance following exercise. The double-blinded, placebo-controlled experiment involved 15 men and 13 women who were untrained in lower-body resistance training. Participants applied approximately 100 mg of either CBD or placebo cream to their quadriceps over three days. The control group underwent a sitting rest period of the same duration as the cream application process. The study found no significant impact of CBD cream use on muscle recovery. Therefore, for those looking to reduce muscle soreness and enhance performance, the current dosage of this topical CBD product may not be effective [67].

Additionally, D’Andre S. et al. (2024) conducted a pilot clinical trial to evaluate the efficacy of a CBD cream (250 mg/3 oz) in treating chemotherapy-induced peripheral neuropathy. Forty patients were randomly assigned to apply either CBD or placebo cream for two weeks, with a crossover design. Neuropathy and side effects were assessed using various tools. The results showed no significant difference in neuropathy or toxicity scores between the CBD and placebo groups. The CBD cream was well tolerated, but the study did not support its effectiveness in improving painful chemotherapy-induced peripheral neuropathy [68].

In a recent review, Cásedas G. et al. (2024) provide comprehensive evidence of CBD’s analgesic and anti-inflammatory properties, highlighting its potential effectiveness in pain management. With its growing therapeutic value in treating osteoarthritis and chronic pain, these effects appear to be primarily mediated through the activation of TRPV-1, 5-HT1A, and CB1 [69].

Concerning the analgesic effect, research on experimental animal models has demonstrated CBD’s ability to modulate pain perception through interactions with various receptors, including CB1, CB2, serotonin receptors (5HT1a), and TRP channels. The combined mechanisms previously described make CBD a potential option for managing neuropathic and inflammatory pain. Clinical studies have shown significant pain relief and decreased disability with topical CBD products, although results vary depending on the condition and dosage. Further research is needed to fully understand CBD’s efficacy and safety in pain management.

### 5.2. Antibacterial Effect

Recent studies in phytotherapy have emphasised the role of cannabis-derived compounds in combating bacterial infections, highlighting an increased interest in their antibacterial properties [70,71].

Appendino G. et al. (2008) examined the antibacterial profiles of the five main cannabinoids, their alkylation and acylation products, a subset of their carboxylic precursors (pre-cannabinoids), and synthetic positional isomers (abnormal cannabinoids) to gather structure–activity data and identify a potential microbiocidal cannabinoid pharmacophore. CBD and other phytocannabinoids (CBC, CBG, CBN, and Δ^9^– THC) exhibited potent activity against various clinically relevant methicillin-resistant *Staphylococcus aureus* (MRSA) strains. The activity was largely unaffected by the prenyl moiety’s nature or position or by the resorcinol moiety’s carboxylation. However, modifications such as methylation and acetylation of phenolic hydroxyls, esterification of the carboxylic group in pre-cannabinoids, and the addition of a second prenyl moiety reduced antibacterial effectiveness. The results suggest that the prenyl moiety primarily modulates the lipid affinity for the olivetol core, which is inherently a weak antibacterial agent. At the same time, the high potency of cannabinoids indicates a specific yet unidentified mechanism of action [72].

Blaskovich M.A.T. et al. (2021) provided a comprehensive evaluation of cannabidiol’s antimicrobial properties, which is the primary non-psychoactive component of cannabis. The findings confirm earlier reports of its activity against Gram-positive bacteria and expand the range of tested pathogens, including highly resistant strains like *Staphylococcus aureus, Streptococcus pneumoniae,* and *Clostridioides difficile*. The results demonstrate that CBD exhibits potent activity against biofilms, has a low likelihood of inducing resistance, and shows effectiveness in topical in vivo applications. Multiple mode-of-action studies suggest that CBD primarily disrupts bacterial membranes. For the first time, CBD is reported to selectively kill a subset of Gram-negative bacteria, including the “urgent threat” pathogen *Neisseria gonorrhoeae*. The structure–activity relationship studies indicate the potential to develop cannabidiol analogues as a new class of antibiotics [11].

Gildea L. et al. (2022) investigated the antibacterial properties of CBD against *Salmonella typhimurium* and *Salmonella newington*. Cultures of *Salmonella typhimurium* and *Salmonella newington* were treated with varying concentrations of CBD, and their optical density (OD600) was measured hourly for 6 h. Both bacteria showed significant reductions in OD600, indicating decreased growth, with *Salmonella newington* being more susceptible to lower CBD concentrations. *Salmonella typhimurium* exhibited increased OD600 at the lowest CBD concentration, suggesting potential resistance development. The study demonstrated CBD’s dose-dependent antibacterial effects on both bacterial strains. Often treated with broad-spectrum antibiotics like ampicillin, *Salmonella* infections are increasingly resistant, necessitating alternative treatments. Comparative kinetic studies showed that both CBD (0.125 µg/mL) and ampicillin (0.5 µg/mL) effectively inhibited the growth of the two *Salmonella* strains. After 6 h, both treatments resulted in similar reductions in OD600, indicating that CBD’s antibacterial efficacy is comparable to ampicillin. It was also noticed that *Salmonella* spp. may have developed resistance to CBD therapy [73].

Additionally, Pillai S.K. et al. (2024) demonstrated that the solvent used to solubilise CBD affects its antimicrobial activity. The antimicrobial properties of solubilised CBD were tested against various microorganisms, including Gram-negative bacteria *(Escherichia coli, Pseudomonas aeruginosa),* Gram-positive bacteria *(Staphylococcus aureus, Staphylococcus, Cutibacterium acnes),* and fungi *(Candida albicans, Malassezia furfur),* using agar well diffusion and broth microdilution techniques. Dissolving CBD in an aqueous solution containing surfactants showed no antimicrobial effect against any of the tested microbes. However, CBD solubilised in an organic medium was ineffective against Gram-negative bacteria but demonstrated excellent antimicrobial activity against the tested Gram-positive bacteria and fungi [74].

The systematic review by Niyangoda D. et al. (2024) highlighted that CBD was the most effective cannabinoid, with MICs between 0.65 and 32 mg/L for *Staphylococcus aureus*, between 0.5 and 4 mg/L for MRSA, between 1 and 2 mg/L for vancomycin-resistant *Staphylococcus aureus* (VRSA), and between 0.6 and 50 mg/L for *Streptococcus pyogenes*. When combined with other antimicrobial treatments, CBD and essential oils from *Cannabis sativa* have been shown to have synergistic effects [71]. Thus, CBD could be a promising candidate for developing new antibacterial therapies [75].

In addition, CBD has been shown to inhibit bacterial proliferation, including that of *Cutibacterium acnes,* a critical factor in the development of acne. This antibacterial property, combined with CBD’s lipostatic, anti-inflammatory, and antiproliferative effects, makes it a promising compound for managing acne [11,12].

The main pharmacophores of CBD related to its antibacterial effect, as identified in the study, are the resorcinol moiety, phenolic hydroxyl groups, the monoterpene region (prenyl moiety), and the aromatic alkyl side chain. The resorcinol moiety is considered the primary antibacterial pharmacophore. The resorcinol structure is crucial for the antimicrobial properties of CBD. Phenolic hydroxyl groups are essential for antibacterial activity; these groups can be free or involved in cyclisation with the monoterpene group. Modifications such as methylation and acetylation of these groups significantly reduce the antibacterial potency. The monoterpene significantly influences both biosynthesis and antibacterial effectiveness. The cyclic form of the monoterpene group, as seen in CBD, enhances antibacterial potency compared to acyclic forms. Additionally, the prenyl group modulates lipid affinity and cellular bioavailability, thereby improving the antibacterial activity of the resorcinol core. The length and position of this chain are crucial for antibacterial activity. The chain typically consists of a 5-carbon structure, and variations in length can affect the efficacy. These pharmacophores collectively contribute to the potent antibacterial effects of CBD against various drug-resistant bacterial strains [71,72].

CBD exhibits antibacterial effects primarily through disrupting bacterial membranes. It causes membrane depolarisation and disrupts the membrane potential in bacteria such as *Staphylococcus aureus*, leading to cell division defects and cell envelope abnormalities. CBD can also block the release of membrane vesicles, affecting bacterial communication. Additionally, CBD inhibits the synthesis of proteins, DNA, and RNA, contributing to its rapid bactericidal action. These mechanisms collectively highlight CBD’s potential in targeting bacterial cell integrity and communication systems [76].

The following key aspects are highlighted based on the discussed studies. CBD exhibit potent antibacterial activity against various drug-resistant bacterial strains, including MRSA, VRSA, and *Salmonella*. CBD’s effectiveness is primarily due to its ability to disrupt bacterial membranes, inhibit protein, DNA, and RNA synthesis, and block the release of membrane vesicles. The antibacterial potency of CBD is influenced by its pharmacophores, including the resorcinol moiety, phenolic hydroxyls, monoterpene region, and aromatic alkyl side chain. CBD has comparable efficacy to conventional antibiotics, such as ampicillin, and can be enhanced when combined with other antimicrobial treatments. CBD’s antibacterial properties also make it a promising candidate for managing acne and other bacterial infections.

### 5.3. Anti-Inflammatory Effect

Numerous studies have highlighted that certain cannabinoids possess significant anti-inflammatory properties, leading to the development of new therapeutic approaches for managing inflammation.

Henshaw F.R. et al. (2021) reviewed several in vivo studies on the effects of cannabinoids on pro- and anti-inflammatory cytokines. Data were extracted from 26 eligible studies in which pro-inflammatory cytokine levels were consistently reduced after CBD, CBG, or CBD combined with Δ^9^–THC treatment, thus exerting anti-inflammatory effects [77]. CBD is also known for its anti-inflammatory effect, presenting, in small quantities, physiological effects that promote and maintain health. The anti-inflammatory effect, combined with the antioxidant effect of CBD and its derivatives, may contribute to protection against oxidative stress and the consequences associated with oxidative modifications of proteins and lipids [58,78].

Sunda F. and Arowolo A. (2020) describe the mechanisms and signalling pathways through which the anti-inflammatory effects of this cannabinoid are observed. These include low affinity for CB1 and CB2 receptors, mimicking the anti-inflammatory effects of endogenous adenosine by acting as an agonist of adenosine A2A receptors, regulating TRPV channels, increasing Ca^2+^ influx through these channels, and inhibiting the secretion of pro-inflammatory cytokines. CBD also influences the balance between the production and elimination of reactive oxygen species (ROS), reducing lipid peroxidation and the oxidation of specific amino acids essential for cellular and tissue homeostasis by decreasing the activity of ROS-producing enzymes, such as NADPH oxidase and increasing the activity of ROS-scavenging enzymes, including superoxide dismutase (SOD). The anti-inflammatory properties also result from the fact that CBD is an agonist of the PPAR-γ receptor, which leads to the inhibition of NF-kB-mediated transcription of pro-inflammatory genes, including the pro-inflammatory cytokines tumour necrosis factor-alpha (TNF-α), IL-1, and IL-6 [59]. Thus, CBD exhibits anti-inflammatory properties by interfering with NF-kB activity and reducing pro-inflammatory gene expression in keratinocytes and dermal fibroblasts [9]. Cannabinoids have demonstrated anti-inflammatory effects by modulating various signalling pathways, including the nuclear factor erythroid 2-related factor 2 (Nrf2) pathway [79].

Additionally, CBD exhibits anti-inflammatory and antioxidant properties. It regulates key factors and receptors to potentially mitigate damage from UV-A exposure. Although it weakly binds to cannabinoid receptors CB1 and CB2, CBD has a significant influence on cell signalling related to inflammation and oxidative stress. CBD offers promising photoprotective benefits, being one of the most effective non-psychotropic cannabinoids against UV-A damage [80].

Several studies were discussed by Ferreira B.P. et al. (2023) regarding the anti-inflammatory effect of CBD. It has been demonstrated that CBD exhibits anti-inflammatory properties by reducing pro-inflammatory cytokine levels, independent of the TRPV4 pathways. CBD achieves this by upregulating TRIB3 via the A2a adenosine receptor and inhibiting NF-kB signalling. Additionally, CBD enhances adenosine signalling, protecting against inflammation by preventing the rapid cellular uptake of adenosine and activating the A2a receptor. Additionally, CBD reduces inflammation caused by extracellular vesicles of Cutibacterium acnes and inhibits the release of pro-inflammatory cytokines, including IL-8 and IL-1, in keratinocytes exposed to *Cutibacterium acnes* [12].

CBD modulates the ECS through the CB2R and TRPV1 pathways and has shown efficacy in animal models of inflammation, as well as in clinical studies on psoriasis, atopic dermatitis, and seborrheic dermatitis (Section 7) [9].

For example, Bunman S. et al. (2024) conducted an in vivo study to explore the anti-inflammatory effects of a 1% topical CBD gel in an animal model. Scientific tests were conducted, including the formalin test, writhing test, and carrageenan-induced oedema evaluation, along with histopathological and proinflammatory mediator analyses. The results demonstrated that the CBD gel significantly reduced inflammation markers—such as paw licking, paw oedema, and writhing response—comparable to or better than diclofenac, a common anti-inflammatory agent, and superior to the placebo. Histopathological findings revealed reduced leukocyte infiltration and inflammation, with plasma proinflammatory mediator levels similar to those observed in individuals treated with diclofenac. Thus, 1% CBD gel is a promising candidate as an anti-inflammatory agent [81].

Based on previous studies, it can be stated that the main possible mechanisms behind the anti-inflammatory effects of CBD are inhibition of the NF-κB pathway, adenosine A2A and PPAR-γ receptor activation, regulation of TRPV channels, downregulation of pro-inflammatory cytokines, reduction in ROS levels, photoprotective and anti-oxidative effects, and modulation of the ECS.

### 5.4. Antioxidant Effect

ROS easily bind to cellular components, causing lipid peroxidation reactions that can lead to cellular damage and, subsequently, the development of malignant diseases. Thus, this can lead to an alteration of the antioxidant defence system, which, under normal conditions, balances peroxidation with the help of the production of glutathione, SOD and catalase, but, in the case of a higher rate of lipid peroxidation, the skin’s endogenous protective antioxidants are not sufficient [82].

Numerous studies have investigated the antioxidant properties of Δ^9^–THC, CBD, synthetic cannabinoids, and extracts from *Cannabis sativa* through various methods. Both Δ^9^–THC and CBD demonstrated antioxidant activity comparable to vitamins E and C. Thus, compared to CBD, Δ^9^–THC exhibited greater antioxidant effectiveness [79]. Borges R.S. et al. (2013) highlighted that CBD has potential antioxidant properties because its cationic free radicals exhibit several resonance structures in which the unpaired electrons are mainly distributed on the ether and alkyl moieties, as well as on the benzene ring [83].

The protective effects of CBD on skin fibroblast models exposed to UVA and UVB radiation were examined in a study by Gęgotek A. et al. (2019). By analysing their proteomic profiles, the researchers evaluated the protective effects of CBD on skin fibroblasts (both 2D and 3D cultures) exposed to UVA and UVB radiation. The study found that the cytoprotective effects of CBD against UV-induced damage varied between 2D and 3D cultured fibroblasts. In 2D cultured cells, UV radiation induced significant changes in protein expression related to antioxidant response and inflammation. In contrast, in 3D-cultured fibroblasts, CBD primarily activated signalling pathways in response to UV-induced changes. This aspect suggests that CBD’s lower protective effect in 3D cultures should be considered when designing UV light protection strategies [10].

Atalay S. et al. (2020) described numerous direct antioxidant effects of CBD, including supporting the action of antioxidant enzymes by preventing the reduction in microelement levels (Zn, Se), directly reducing the level of oxidants, modifying the redox balance by changing the level and activity of antioxidants, reducing ROS and oxidative modifications of lipids, proteins, and DNA [58].

The antioxidant effects of cannabinoids are likely related to their chemical structure. Structural features that contribute to their oxidising capacity are phenolic groups and double bonds. Phenolic groups can donate electrons or hydrogen atoms to free radicals, thus neutralising them and preventing oxidative damage to cells and DNA. Double bonds can participate in chemical reactions that help neutralise reactive oxygen species and prevent oxidative processes. In a study conducted by Dawidowicz et al. (2021), CBD and six other cannabidiols were examined using spectrophotometric methods. As a result, the cannabinoids demonstrated antioxidant activity, characterised by their ability to neutralise free radicals, prevent oxidation processes, and reduce metal ions [84].

CBD and Δ^9^–THC exhibit antioxidant properties by influencing oxidative stress regulators (Nrf2 and BACH1) and redox balance. They act by interrupting the production of free radicals, facilitating mitochondrial repair, and enhancing the expression of antioxidant enzymes. Their ability to protect against oxidative damage, neurotoxicity, and inflammation often surpasses that of traditional antioxidants, such as vitamins E and C. Additionally, their effects appear independent of cannabinoid receptors [79].

It is well known that oxidative stress damages the skin, leading to inflammation and premature ageing. Thereby, CBD protects skin cells by reducing ROS, enhancing antioxidant systems, increasing vitamin A and E levels, and preventing lipid peroxidation. CBD also activates the antioxidant enzyme heme oxygenase 1 by degrading its negative regulator, BACH1, and modulates other key factors, such as PPAR-γ and NF-κB, suggesting its protective role against oxidative stress [9,10,79,84].

Summarising the data presented above, CBD exhibits significant antioxidant properties by neutralising ROS and preventing oxidative damage to cellular components such as lipids, proteins, and DNA. It has been demonstrated that CBD can support antioxidant enzymes, control the reduction in essential microelements such as zinc and selenium, and modulate the redox balance. Additionally, CBD influences oxidative stress regulators such as Nrf2 and BACH1, enhances mitochondrial repair, and increases the expression of antioxidant enzymes. The listed mechanisms help protect against oxidative damage, neurotoxicity, and inflammation, often performing as well as or better than traditional antioxidants like vitamins E and C. CBD’s protective effects against UV-induced damage in skin fibroblasts further highlight its potential in skincare and anti-ageing applications.

### 5.5. Antiproliferative Effect

Cannabinoids, particularly CBD and Δ^9^–THC, have garnered increasing attention for their antiproliferative and pro-apoptotic properties. These compounds exert multifaceted effects on tumour biology, including inhibition of cell proliferation, induction of apoptosis and autophagy, suppression of angiogenesis, and modulation of chemoresistance. CBD, in particular, interacts with a variety of molecular targets beyond the classical cannabinoid receptors, influencing pathways such as the PI3K/AKT/mTOR, MAPK/ERK, and NF-κB pathways. Its potential as an anti-cancer agent and its support for an emerging role in dermatological applications, including the treatment of skin cancers and acne, arise from these mechanisms [85].

#### 5.5.1. Mechanisms of Action

CBD exhibits anti-cancer properties by inhibiting cell proliferation, inducing apoptosis, and suppressing tumour angiogenesis. These effects involve multiple pathways, including interactions with the ECS through CB1 and CB2 receptors, as well as other receptors like GPR55, TRPV1, and PPARs. The interactions modulate cancer-related signalling pathways, leading to the inhibition of cell growth, induction of apoptosis, and suppression of tumour angiogenesis. Also, CBD is effective in promoting autophagy and apoptosis in cancer cells, highlighting its potential as an anti-cancer agent [85,86].

Additionally, CBD influences the redox balance and regulation of oxidative stress, including the activation of Nrf2 and BACH1, which contribute to its antiproliferative effects. Also, CBD’s ability to reduce chemotherapy-induced nausea and vomiting, along with its analgesic effects, further supports its use in cancer therapy [86]. Understanding the redox-modulatory activity of cannabinoids in cancer treatment is essential. Future research should also investigate the interaction between Nrf2’s anti-inflammatory capabilities in normal cells and the inflammatory tumour microenvironment by examining free radical scavenging, metal ion reduction, and protection against oxidative processes [79].

Several cannabinoids, including CBD, are proposed to inhibit the proliferation of keratinocytes in a concentration-dependent manner, regardless of whether cannabinoid receptor activation occurs. Various inhibition mechanisms are likely, such as activating the CB1 receptor by a specific agonist, leading to a reduction in keratin K6 and K16 expression, which have also been observed [3,28].

#### 5.5.2. Evidence from Preclinical Studies

Numerous preclinical studies have demonstrated CBD’s ability to inhibit tumour cell proliferation, invasion, metastasis, and angiogenesis. For example, CBD has been shown to have cytotoxic effects on various cancer cell lines, including those of colorectal, breast, and glioblastoma cells. The dual antiproliferative and proapoptotic effects of cannabinoids have been studied across multiple cancer cell lines. Additionally, cannabinoids demonstrate strong anti-cancer activity against tumour xenografts, including those resistant to standard chemotherapies [87].

In melanoma models, CBD and its derivatives have demonstrated enhanced cytotoxicity and induction of apoptosis. The antiproliferative effects of various phytocannabinoids were evaluated on murine (B16F10) and human (A375) melanoma cells, with CBD exhibiting the highest cytotoxicity (IC50 values of 28.6 and 51.6 μM, respectively). Further testing on B16F10 cells revealed two synthetic CBD derivatives (22 and 34) with significantly enhanced cytotoxicity (IC50 of 3.1 and 8.5 μM, respectively). Cell death assays, including flow cytometry for apoptosis and ferroptosis and lactate dehydrogenase for pyroptosis, were used to analyse the antiproliferative effects of CBD and its derivatives. The increased cytotoxicity of two bipiperidinyl derivatives was linked to their ability to induce apoptosis. CBD and its derivatives are promising candidates for cannabinoid-based melanoma treatments [88].

Also, a cannabinoid solution with a Δ^9^–THC to CBD ratio of 1:6 has the potential to inhibit cell proliferation and induce apoptosis in human pancreatic ductal adenocarcinoma xenograft models [89].

Sainz-Cort A. et al. (2020) evaluated the viability and proliferation of cancer cells treated with CBD in the presence of 10% serum, which typically supports cell growth. The findings revealed that CBD had a cytotoxic effect on the human HT-29 cancer cell line only in the presence of 0.5% serum, but not in the presence of 10% serum. In 10% serum, CBD did not inhibit DNA replication in HT-29 cells and showed only weak inhibition in other cancer cell lines. These results suggest that CBD’s effects are cell context-dependent and influenced by the presence of growth factors [90].

Lee, H.-S. et al. (2022) found that CBD reduces the viability of human colorectal cancer cells in a dose-dependent manner, causing G1-phase cell cycle arrest and increasing the apoptotic cell population. CBD downregulated cyclin proteins and CDKs, increased caspase activity, and elevated ER stress proteins. The mechanism of CBD-induced cell death was dependent on CB2 receptors, but not on CB1, TRPV, or PPAR-γ receptors [91].

The study conducted by D’Aloia A. et al. (2022) on the biological effects of CBD on MDA-MB-231 cells reveals that both CBD dosage and serum concentrations significantly influence outcomes. Serum acts as a surfactant, affecting CBD aggregation. CBD shows protective effects against cisplatin’s cytotoxicity under standard conditions. In low serum conditions (0.5%), CBD forms aggregates at concentrations above 5 µM, resulting in cytostatic effects, cell cycle arrest, and activation of autophagy. Higher doses induce cytotoxicity and bubble cell death. IGF-1 and EGF counteract CBD’s antiproliferative effects, highlighting the importance of CBD’s physical state and concentration in its biological impact [92].

CBD has shown promising anti-cancer effects in vitro and in vivo studies by inhibiting tumour growth, inducing cell death, and reducing metastasis in various cancer types. Its effectiveness varies depending on the cancer cell type, serum conditions, and presence of growth factors, with some synthetic derivatives showing enhanced potency. These findings support CBD’s potential as a complementary cancer therapy, especially in melanoma and colorectal cancers.

#### 5.5.3. Evidence from Clinical and Dermatological Studies

Although some studies have explored the synergistic potential of cannabinoids with conventional oncology treatments, based on the concept of “cannabinoid sensitisers”, clinical trials assessing their antineoplastic effects have involved only a limited number of subjects, resulting in no definitive conclusions about their efficacy [87]. For example, CBD derivatives with improved antiproliferative properties could work synergistically with chemotherapy drugs, offering potential for developing cannabinoid-based adjuvant therapies to treat ovarian cancer [93].

Non-melanoma skin cancers, including basal cell carcinoma and squamous cell carcinoma, are the most prevalent malignancies in humans. Due to the growth-inhibitory effects of cannabinoids on key receptors, extensive research has been conducted on their potential as anti-tumour therapies. Cannabinoid extracts have demonstrated benefits in treating non-melanoma skin cancers by inducing apoptosis, inhibiting tumour angiogenesis, and arresting the cell cycle. Additionally, cannabinoids have therapeutic effects on melanomas, likely through cell cycle arrest, apoptosis, and other less-understood mechanisms [63].

More clinical studies are needed to fully understand and validate the efficacy of CBD in cancer treatment [86].

#### 5.5.4. Use of Cannabidiol in Dermatological Conditions

The antiproliferative effect of cannabinoids could be helpful in various dermatological conditions, including non-melanoma skin cancers, melanomas, and acne [3,63].

Abassi-Rana G. et al. (2024) found that CBD combined with temozolomide, fluoxetine, and doxorubicin significantly reduced U87-MG cell viability in vitro, with an even stronger effect observed between CBD and temozolomide in GIN-8 cells. Thus, CBD could serve as an anti-cancer drug for both core and invasive margin cells. Due to the diverse nature of glioblastoma, further research is needed to understand the molecular mechanisms behind CBD’s anti-tumour effects and to assess its potential integration into existing treatments [94].

Also, CBD is considered a promising treatment for acne vulgaris due to its ability to regulate sebocyte cell lipogenesis without affecting cell viability, reduce sebocyte cell proliferation without causing apoptosis, and lower pro-inflammatory cytokine levels, providing anti-inflammatory benefits [95,96].

Antiproliferative effects are vital in addressing acne vulgaris by targeting the abnormal growth and replication of sebocytes. These effects limit the rapid division of sebocytes, thereby controlling excessive sebum production. CBD helps regulate sebocyte activity, curbing their overgrowth. This regulation helps restore balanced sebum production, essential for effective acne treatment [97].

CBD shows strong potential in dermatological applications due to its antiproliferative and anti-inflammatory properties. It may aid in treating conditions like acne and skin cancers by regulating cell growth and reducing inflammation. Further research is needed to understand its mechanisms and fully optimise its clinical use, although promising results have been observed, especially in combination therapies for glioblastoma. In conclusion, CBD demonstrates significant antiproliferative activity across various cancer models, including skin-related malignancies. Its ability to modulate key signalling pathways and induce apoptosis positions it as a promising candidate for adjunctive cancer therapies and dermatological applications. However, further clinical trials are essential to validate these effects in humans.

### 5.6. Lipostatic Effect

Cannabinoids, particularly CBD, have shown lipostatic effects, meaning they can regulate lipid production. The lipostatic effects are particularly relevant in the context of skin health and conditions like acne. CBD has been found to modulate sebum production, reduce inflammation, and inhibit the proliferation of sebocytes (the cells that produce sebum). These properties make cannabinoids promising candidates for treating acne and other skin disorders characterised by excessive sebum production [12,26,96,98].

CBD and palmitoylethanolamide (PEA) have therapeutic potential for inflammatory skin conditions such as atopic dermatitis and acne vulgaris due to their anti-inflammatory and lipostatic effects. CBD inhibits the lipogenic actions of various compounds and suppresses sebocyte proliferation by activating TRPV4 ion channels [26].

Also, topical cannabinoids are recognised for their lipostatic properties in small-scale human preclinical studies and animal models [12,98]. Cannabichromene (CBC) and cannabidivarin (CBDV) exhibited considerable lipostatic effects, but tetrahydrocannabivarin (THCV) proved to be the most promising anti-acne agent. CBD and THCV showed comprehensive lipostatic actions, effectively countering ‘acne-mimicking’ activity triggered by substances through separate pro-lipogenic signalling pathways [96].

Activation of peroxisome proliferator-activated receptor α (PPAR-α) by PEA may lead to the downregulation of genes involved in lipogenesis (the synthesis of new lipids) and stimulate fatty acid oxidation, which helps reduce lipid accumulation in adipose tissues [25].

Recently, Ferreira I. et al. (2024) discussed the lipostatic effect of cannabinoids, particularly CBD, in the context of acne treatment. CBD has been shown to regulate sebum production, which is a critical factor in the development of acne. By modulating sebum production, CBD helps reduce excess skin oiliness, thereby preventing the formation of acne lesions. This lipostatic effect, combined with CBD’s anti-inflammatory, antiproliferative, and antimicrobial properties, makes it a promising compound for acne treatment [12].

Thus, CBD exhibits strong lipostatic, anti-inflammatory, and antiproliferative effects. Also, CBD regulates lipid synthesis, reduces sebocyte proliferation, and activates TRPV4 channels to suppress oil production. Other cannabinoids like THCV and CBC also demonstrated potential, with THCV emerging as a particularly effective anti-acne compound, highlighting the therapeutic potential of cannabinoids in dermatology.

### 5.7. Melanogenesis Impairment (Skin, Hair)

The ECS in human melanocytes involves CB1, CB2, and transient receptor potential vanilloid 1 (TRPV-1) receptors. Endocannabinoids induce apoptosis in melanocytes through TRPV-1 at high concentrations, leading to increased DNA fragmentation and p53 expression. Conversely, they stimulate melanin production and tyrosinase activity at low concentrations through CB1 receptors. Thus, endocannabinoids can both promote melanogenesis at low levels (helpful in treating depigmentation disorders) and induce apoptosis at high levels (potentially beneficial for treating melanomas and melasma) [63]. However, few studies show how phytocannabinoids, such as CBD, affect melanogenesis [8].

CBD promotes melanogenesis by activating specific signalling pathways and increasing the expression of key melanogenic genes through the CB1 receptor. It enhances melanin production by upregulating the expression of the microphthalmia-associated transcription factor (MITF) and tyrosinase, which are crucial for melanin synthesis. This process involves the activation of p38 MAPK and p42/44 MAPK signalling pathways. Additionally, CBD’s effect on melanogenesis is independent of the cAMP-PKA signalling pathway [97,99,100].

Some relevant studies are briefly summarised further. Hwang Y.S. et al. (2017) investigated the impact of CBD on melanogenesis, the process of melanin production, in human epidermal melanocytes. The findings reveal that CBD enhances both melanin content and tyrosinase activity. It also upregulates the mRNA and protein levels of MITF, tyrosinase, tyrosinase-related protein 1 (TRP-1) 1, and tyrosinase-related protein 2 (TRP-2). Mechanistically, CBD stimulates melanogenesis by phosphorylating p38 MAPK and p42/44 MAPK, independent of cAMP-PKA signalling. Notably, the melanogenic effect of CBD is mediated through the CB1 receptor, rather than the CB2 receptor. These results suggest that CBD-induced melanogenesis is CB1 receptor-dependent and involves increased MITF gene expression, mediated by the activation of p38 MAPK and p42/44 MAPK. Consequently, CBD may protect against external stresses such as ultraviolet irradiation and oxidative stressors [99].

Goenka, S. (2022) examined the impact of CBD on melanogenic activity in B16F10 melanoma cells. CBD treatment at concentrations of 0, 1.25, and 2.5 µg/mL increased melanin content in a dose-dependent manner. Additionally, qPCR analysis showed that CBD significantly enhanced the expression levels of tyrosinase, a key gene involved in melanogenesis [101]. Also, Kim T. et al. (2023) found that CBD has an anti-melanogenic effect. When melan-a cells were treated with *Cannabis sativa* stem extracts fermented with *Weissella paramesenteroides* (CSWP), there was a significant reduction in melanin content. Additionally, CSWP downregulated the expression of several melanogenesis factors, including TRP-1 and TRP-2, suggesting that CBD, through CSWP, can reduce melanin production by inhibiting the expression of key melanogenesis-related proteins [102].

In addition, Tassaneesuwan N. et al. (2023) investigated the effect of CBD on melanogenic activity by measuring melanin content in B16F10 melanoma cells treated with CBD at three concentrations (0, 1.25, and 2.5 µg/mL). The results showed that CBD increased melanin content in a dose-dependent manner. Additionally, qPCR analysis revealed that CBD significantly enhanced the expression levels of tyrosinase, a key melanogenic-related gene, in the treated cells [103].

The work of Gaweł-Bęben K. et al. (2023) explored the in vitro anti-melanoma, anti-melanogenic, and anti-tyrosinase effects of CBD and other minor phytocannabinoids (CBG, CBN, and CBC). CBD significantly decreased extracellular and intracellular melanin content in α-melanocyte-stimulating hormone (αMSH)-treated murine melanoma B16F10 cells. However, CBD was practically inactive in inhibiting tyrosinase activity, suggesting that the reduction in melanin biosynthesis is not due to tyrosinase inhibition; this indicates that CBD’s role in melanogenesis involves mechanisms other than tyrosinase inhibition [100].

The essential key points are presented based on several published studies that have explored the effects of CBD on melanogenesis, as previously discussed. CBD enhances melanin content and tyrosinase activity in human epidermal melanocytes, a process mediated through CB1 receptors and involving the p38 MAPK and p42/44 MAPK pathways. Additionally, CBD increases melanin content in B16F10 melanoma cells in a dose-dependent manner. CBD’s dose-dependent increase in melanin content and tyrosinase expression in B16F10 cells was confirmed. Thus, through CSWP, CBD reduces melanin production by downregulating the factors involved in melanogenesis. Also, CBD decreases melanin content in αMSH-treated murine melanoma cells without inhibiting tyrosinase activity, suggesting alternative mechanisms.

### 5.8. Moisturising Effect

Several studies have highlighted the moisturising effect and the mechanisms by which it occurs.

For example, relevant research on the moisturising properties of CBD and its mechanism was conducted by Ikarashi N. et al. (2021). A 1% CBD solution was applied daily to the skin of seven-week-old male HR-1 hairless mice for 14 days. The dermal water content in the CBD-treated mice significantly increased compared to the control group. Additionally, no inflammatory reactions or obvious skin disorders were observed. The mRNA expression levels of loricrin, filaggrin, collagen, hyaluronic acid-degrading enzyme, hyaluronic acid synthase, ceramide-degrading enzyme, and ceramide synthase in the skin were unaffected by CBD application. However, aquaporin-3 (AQP3), a member of the aquaporin family, showed significantly higher levels in the CBD-treated group at both the mRNA and protein levels. This indicates that CBD moisturises the skin, potentially due to the increased expression of AQP3, which plays a crucial role in skin water retention. CBD is anticipated to be developed as a cosmetic ingredient with a unique mechanism [104].

Additionally, the review by Ferreira B. et al. (2023) explores the therapeutic potential of CBD for various skin conditions, including its hydrating and moisturising properties. It discusses how CBD can reduce excessive skin water loss and increase its water content. CBD enhances skin moisture by reducing excessive water loss through multiple mechanisms. It stimulates sphingomyelinase, increases ceramide levels, reinforces the epidermis structure, and preserves skin moisture. Additionally, CBD boosts the expression of AQP3, improving glycerol transport and skin water retention. Studies on hairless mice have shown that CBD increases dermal water content (DWC) while maintaining skin barrier integrity. The moisturising effect is primarily due to AQP3 modulation, likely through PPAR-γ activation [97]. No change in Pparg mRNA levels was observed in CBD-treated mouse skin [9]. Furthermore, CBD reduces sphingomyelin concentration and increases sphingomyelinase activity, thereby raising ceramide levels, which are crucial for maintaining the transepidermal barrier function and preventing excessive water loss. Thus, CBD promotes skin water retention and improves DWC [9,97].

In addition, Kuzumi A. et al. (2024) claim that DWC is crucial for maintaining skin integrity, as its loss can lead to reduced elasticity, wrinkle formation, and ageing. Topical application of CBD has been shown to significantly increase DWC, accompanied by an increase in AQP3, which is essential for skin water retention. Additionally, CBD increased the expression of filaggrin and involucrin in keratinocytes and epidermal equivalents. Clinical studies also support CBD’s moisturising properties, showing decreased transepidermal water loss (TEWL) and improved skin hydration and elasticity [9,105,106].

In summary, CBD has been shown to increase DWC and improve skin hydration and elasticity significantly. The application of the CBD solution increased DWC due to the increased expression of AQP3 (in hairless mice). Additionally, CBD stimulates sphingomyelinase, leading to higher ceramide levels that reinforce the epidermis structure and preserve skin moisture. Clinical studies also support CBD’s moisturising properties, showing decreased TEWL and improved skin hydration and elasticity. Further research is needed to fully understand the mechanisms behind CBD’s moisturising effects and the receptors involved.

### 5.9. Skin Protection

Due to their anti-inflammatory and antioxidant properties, as well as their ability to modulate the skin’s immunological and neuroendocrine responses, cannabinoids are recognised as skin-protecting agents. To preserve skin homeostasis and provide protection against various dermatological conditions, active ingredients from *Cannabis sativa* L., such as CBD, interact with the skin’s ECS. For instance, by preventing aberrant cell proliferation, lowering inflammation, and promoting melanin synthesis, cannabinoids have shown therapeutic promise in treating dermatitis, psoriasis, acne, and even skin cancer [3].

For example, the study by Gęgotek A. et al. (2019) highlighted how CBD protects the skin by examining its effects on fibroblasts exposed to UVA and UVB radiation. The study found that CBD treatment reduced the release of ROS and decreased the expression of matrix metalloproteinase (MMP) enzymes, which are responsible for degrading connective tissue; this led to increased collagen expression and reduced the formation of harmful adducts between lipid peroxidation products and proteins. Overall, CBD enhances the signalling of endogenous antioxidants, providing protective effects against UV-induced skin damage [10].

The stratum corneum lipids, mainly ceramides, free fatty acids, and cholesterol, are essential for skin barrier function. An imbalance in these lipids can lead to skin repair, dryness, and permeability problems. The absence of CB1 delays barrier recovery, while the lack of CB2 accelerates it. The ECS also influences the secretion of lamellar bodies and the expression of late differentiation marker proteins, such as filaggrin, loricrin, and involucrin; this suggests that cannabinoid antagonists may play a key role in enhancing skin barrier repair [63].

Baswan S.M. et al. (2020) highlighted several mechanisms by which CBD can protect the skin. CBD has demonstrated the ability to induce the expression of Hemeoxygenase 1 (HMOX1) and other Nrf2-regulated genes, which play crucial roles in the cellular antioxidant defence system. This induction helps counteract oxidative stress, which can lead to cell damage and chronic inflammation. Additionally, CBD has been shown to penetrate human keratinocytes, balancing oxidative stress responses triggered by UVB irradiation and hydrogen peroxide, and protecting membrane integrity by preventing the reduction in polyunsaturated fatty acids. CBD also activates PPAR-γ, which, along with HMOX1, contributes to its anti-inflammatory, antioxidant, and anti-apoptotic properties. These effects are beneficial for skin conditions characterised by inflammation and keratin disorders, such as eczema or atopic dermatitis. CBD’s ability to modulate oxidative stress and inflammation makes it a promising candidate for protecting and maintaining healthy skin.

McCormick E. et al. (2024), as a result of their pilot clinical trial, highlight that CBD possesses anti-inflammatory and antioxidant properties by regulating nuclear erythroid 2-related factor, HMOX1, and PPAR-γ activity, making it a promising candidate for mitigating UV-A exposure damage. Although it weakly binds to cannabinoid receptors CB1 and CB2, CBD has a significant influence on cell signalling pathways related to inflammation and oxidative stress. The photoprotective benefits of CBD are encouraging, with one comparative study identifying it as the most effective non-psychotropic cannabinoid against UV-A damage [80].

Regarding the protective effect of CBD, based on the identified studies, we can state that CBD could prevent abnormal cell proliferation, reduce inflammation, and promote melanin synthesis. CBD protects skin fibroblasts from UVA and UVB radiation, thereby increasing collagen production and reducing the formation of harmful adducts. Additionally, CBD enhances the signalling of endogenous antioxidants, protecting against UV-induced skin damage. The ECS also plays a crucial role in skin barrier function, with CB1 and CB2 receptors influencing lipid balance and the expression of differentiation marker proteins. CBD’s ability to modulate oxidative stress and inflammation makes it a promising candidate for maintaining healthy skin and treating various skin disorders.

## 6. Cannabidiol Skin Delivery Systems

For dermatological and cosmetic applications, topical and transdermal routes are preferred to achieve high local concentrations with minimal systemic side effects, thereby avoiding issues such as acid degradation and first-pass metabolism. Most topically applied CBD remains on the skin surface, which limits its efficacy in reaching deeper skin layers where therapeutic action is needed. Due to its highly lipophilic nature (Table 2), delivering CBD through the stratum corneum to deeper skin layers necessitates specialised delivery systems. Its poor water solubility and susceptibility to degradation further complicate effective topical or transdermal delivery. To enhance transdermal CBD delivery, chemical penetration enhancers modify the stratum corneum’s lipid domain, improving delivery efficiency [9,97]. For example, oleic acid-enhanced formulations serve as chemical penetration enhancers, significantly improving CBD delivery to the epidermis, particularly in conditions such as psoriasis and atopic dermatitis [107]. Also, a study conducted by Lapteva M. et al. (2024) compared various CBD formulations and found that colloidal systems, especially CG 1% (contains CBD in a concentration of 1%), delivered CBD more effectively to key skin layers than commercial products, making them more suitable for dermatological treatments [108].

Researchers have combined CBD with various substances (e.g., boswellic acid, hyaluronic acid, and volatile oils). Encapsulating CBD in nanoparticulate carriers, such as liposomes and niosomes, has also shown potential. Physical enhancement methods, such as microneedles and ultrasound transport, may further improve CBD delivery [9,97,109].

Therefore, innovative delivery technologies are crucial for enhancing the bioavailability of CBD and ensuring consistent therapeutic outcomes [108]. Table 3 summarises various advanced delivery systems developed to improve the topical and transdermal administration of CBD, highlighting their mechanisms, advantages, and limitations. However, further studies are needed to overcome the challenges of low skin penetration.

## 7. Therapeutic Potential of Cannabidiol in Dermatology

**Figure 6:**
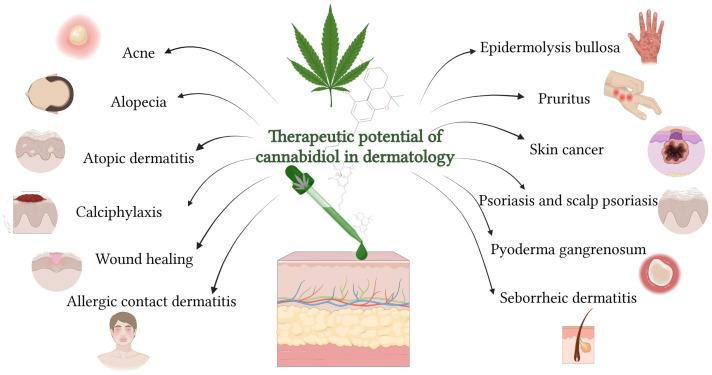
Potential of cannabidiol (CBD) in dermatology (created with BioRender.com).

Considering CBD’s pharmacological profile and its interaction with the cutaneous ECS, it further provides a clinically oriented overview of its therapeutic applications in dermatology (Figure 6), supported by experimental and clinical evidence across a broad spectrum of skin disorders. Currently, five clinical trials are investigating the use of CBD in dermatology. These studies focus on:

- Acne vulgaris (NCT06362889, a phase I study on microneedling with CBD and hempseed oil) (https://clinicaltrials.gov/study/NCT06362889, accessed on 26 June 2025).
- Atopic dermatitis (NCT06022874, an observational study on topical CBD products) (https://clinicaltrials.gov/study/NCT06022874, accessed on 26 June 2025).
- Severe pruritus (NCT06435299, a phase III study on CBD oil) (https://inclinicaltrials.com/pruritus/NCT06435299/, accessed on 26 June 2025).
- Scar healing (NCT05650697, phase I studies on topical CBD) (https://clinicaltrials.gov/study/NCT05650697, accessed on 26 June 2025).
- Scar healing (NCT06129591, phase I studies on topical CBD) (https://clinicaltrials.gov/study/NCT06129591, accessed on 26 June 2025) [9].

### 7.1. Acne

Acne vulgaris is a common dermatological condition that affects millions worldwide, significantly impacting their quality of life. Due to its multifactorial nature, innovative and effective treatment strategies are essential. Recently, there has been growing interest in natural topical therapies, with cannabinoids emerging as promising compounds for research. Cannabinoids are particularly noteworthy in acne treatment due to their lipostatic, anti-inflammatory, antiproliferative, and antimicrobial properties. Among them, CBD stands out for its broad therapeutic potential. Pre-clinical and clinical studies have demonstrated CBD’s ability to regulate sebum production, reduce inflammation, and inhibit bacterial growth, all of which are crucial factors in the development of acne [12].

Also, Yoo E.H. and Lee J.H. (2023) described CBD as a promising therapeutic agent for acne due to its multifaceted actions, including anti-inflammatory, anti-lipogenic, and antimicrobial effects. While these findings are encouraging, the authors emphasise the need for further clinical studies to validate CBD’s efficacy and safety in treating acne [25].

Acne is a common skin condition linked to excessive sebum production and inflammation of sebaceous glands, where components of the ECS, including CB1R, CB2R, and related enzymes, are active. CBD has been shown to inhibit lipid production and sebocyte proliferation by activating TRPV-4 channels, which disrupts the ERK1/2 MAPK pathway, and to reduce inflammation through A2A adenosine receptor activation and NF-κB pathway inhibition [25,95].

CBD also counteracts inflammation caused by *Cutibacterium acnes*—a bacterium associated with acne—by reducing cytokine expression via CB2R activation and TRPV-1 antagonism [11,113,114]. Optimal CBD concentrations (1–10 μM) reduce sebocyte proliferation, with higher doses inducing apoptosis, and concentrations of 5–10 μg/mL effectively inhibit *Cutibacterium acnes* growth [95,115]. A 5% CBD formulation (BTX 1503) is currently undergoing clinical evaluation, with preliminary results indicating that it is safe and well-tolerated in patients with moderate to severe acne [116].

### 7.2. Allergic Contact Dermatitis

A comprehensive review published by Martins A.M. et al. (2022) highlights the therapeutic potential of cannabinoids, especially CBD, in treating inflammatory skin diseases, including allergic contact dermatitis. The review emphasises that topical CBD formulations have shown promising anti-inflammatory effects and are generally well tolerated. However, it also notes that more controlled human clinical trials are needed to confirm efficacy and safety in allergic contact dermatitis specifically [28].

Jiang Z. et al. (2022) explored the effects of CBD on keratinocytes exposed to inflammatory stimuli. Although the primary focus was on acne, the mechanisms—such as suppression of IL-6, IL-8, and TNF-α via CB2 receptor activation and TRPV-1 modulation—are directly relevant to allergic contact dermatitis, where similar inflammatory pathways are involved [114].

Yoo E.H. and Lee J.H. (2023) explored the role of cannabinoids, particularly CBD, in managing skin diseases, including allergic contact dermatitis. CBD interacts with the skin’s ECS (specifically CB2 receptors and other targets like TRPV-1 and PPAR-γ) to modulate immune responses and reduce inflammation. In allergic contact dermatitis, which is driven by T-cell-mediated immune activity, CBD has been shown to suppress inflammatory cytokines, such as TNF-α and IFN-γ, reduce mast cell and T-cell infiltration, and alleviate the severity of skin lesions. The reviewed studies suggest that CBD holds therapeutic potential for allergic contact dermatitis due to its multi-targeted anti-inflammatory and immunosuppressive effects. Unlike corticosteroids, CBD may offer a safer alternative with fewer side effects, making it a promising candidate for topical treatment in inflammatory skin conditions [25].

A recent study conducted by Wolińska R. et al. (2025) found that a CBD-rich cannabis extract outperformed hydrocortisone in reducing skin inflammation in a mouse model. The extract lowered inflammatory markers and exhibited antioxidant activity, indicating strong potential for dermatological applications. When applied topically in the form of an extract-rich ointment, CBD shows promising anti-inflammatory and immunomodulatory effects in a rat model of allergic contact dermatitis. While it may not relieve itching, it could serve as a natural alternative or adjunct to corticosteroids for managing inflammation in allergic contact dermatitis [117].

### 7.3. Alopecia

There is currently limited clinical evidence directly supporting the use of CBD for alopecia treatment. Most existing data focuses on general wellness or unrelated medical conditions. However, interest in CBD as a potential therapy for hair loss is growing, particularly due to the limitations and side effects of conventional treatments. CBD interacts with the body’s ECS, particularly CB1 and CB2 receptors, which are involved in regulating hair follicle function. This interaction suggests a possible mechanism for influencing hair growth when CBD is applied topically or taken orally [118].

A study involving 35 individuals with androgenetic alopecia assessed the effects of a daily topical hemp oil formulation containing 3–4 mg of CBD over six months. The results showed a statistically significant 93.5% increase in hair count in the most affected areas, with men and the vertex region responding more favourably. Importantly, no adverse effects were reported. These findings suggest that CBD may offer a complementary approach to existing treatments like finasteride and minoxidil, potentially enhancing outcomes through synergistic mechanisms [25,119].

CBD’s effects on hair growth appear to be dose-dependent in the study conducted by Smith C.L. and Satino G. (2021) [119]. While lower concentrations may promote regrowth, higher doses (≥10 μM) could induce hair loss by prematurely triggering the catagen phase via TRPV-4 receptor activation. Additionally, other phytocannabinoids present in hemp extracts may counteract CBD’s benefits, underscoring the importance of formulation purity. The topical preparation used in the study was formulated from ultra-pulverised hemp flower infused into a lanolin and emu oil base, highlighting the need for standardised, well-characterised products [119,120].

CBD acts as a partial antagonist of the CB1 receptor, which has been associated with hair shaft elongation. It also activates the TRPV-1 and TRPV-4 receptors, which regulate the phases of the hair cycle. Furthermore, CBD enhances Wnt signalling, a pathway critical for initiating new hair follicle formation and maintaining the anagen (growth) phase. These multifaceted actions highlight CBD’s potential as a novel therapeutic agent for alopecia [121].

More rigorous preclinical and clinical studies are necessary to confirm the efficacy and safety of CBD in alopecia treatment. Future research should focus on identifying optimal dosing, minimising the presence of other cannabinoids, and establishing standardised formulations for consistent therapeutic outcomes [118,120].

### 7.4. Atopic Dermatitis

Affecting up to 20% of children and 3% of adults worldwide, atopic dermatitis, also known as atopic eczema, is a chronic inflammatory skin disease characterised by red, itchy skin lesions, dry skin, scaly patches, and skin pain. The intensity of itching can range from mildly annoying to completely debilitating, significantly impacting patients’ quality of life. Atopic dermatitis can lead to social challenges, detachment, and stigmatisation. Furthermore, this condition is linked to sleep issues, anxiety, hyperactivity, and depression. Although the underlying pathological process is not fully understood, it appears to be a mix of skin barrier dysfunction and immune system irregularities. Commonly, topical emollients are used to repair the skin barrier, but they often yield only limited clinical benefits [122,123].

CBD exerts therapeutic effects in atopic dermatitis by modulating the ECS, a complex signalling network present in the skin. CBD indirectly enhances ECS activity by inhibiting FAAH, the enzyme responsible for breaking down anandamide, thereby increasing its anti-inflammatory and analgesic effects. Additionally, CBD interacts with non-cannabinoid receptors, such as TRPV-1, which are involved in pain and itch signalling, and GPR55, contributing to immune modulation and skin barrier restoration [36,124]. Blocking CB1 and CB2 receptors may also produce pain-relieving and anti-inflammatory effects, potentially because endocannabinoids (as CBD) can act as both promoters and suppressors of inflammation depending on the context [125]. These mechanisms collectively support CBD’s potential to reduce inflammation, alleviate pruritus, and improve skin homeostasis in individuals with atopic dermatitis.

Emerging evidence suggests that CBD may be effective in managing symptoms of atopic dermatitis [25,123]:

- Anti-inflammatory properties: CBD has been shown to have anti-inflammatory effects, which can help reduce the inflammation associated with atopic dermatitis (Section 5.3);
- Anti-itch and anti-pain properties: CBD may help alleviate itching and pain, which are common symptoms of atopic dermatitis (Section 5.1 and Section 5.3);
- Antibacterial effects: Some studies suggest that CBD has antimicrobial properties, which can help prevent infections that often accompany atopic dermatitis (Section 5.5);
- Skin barrier improvement: CBD may help improve the skin barrier function, which is often compromised in individuals with atopic dermatitis (Section 5.9).

Some relevant studies are briefly presented below.

Palmieri B. et al. (2019) conducted a small-scale clinical study involving 20 patients with various skin conditions, including 5 with atopic dermatitis. Participants applied a CBD ointment twice daily for three months. The study reported improvements in skin hydration, elasticity, and symptom relief, with no adverse reactions observed. The authors concluded that CBD ointment is a safe and effective non-invasive treatment for certain inflammatory skin disorders [106].

The study conducted by Maghfour J. et al. (2020) investigated the effects of CBD on individuals with self-reported eczema. Twenty participants consented, and sixteen completed a 28-item online questionnaire assessing disease severity (POEM) and emotional burden (QOLHEQ). The study found significant reductions in disease severity and improvements in emotional well-being. Specifically, 67% reported a decrease in itching, and 50% perceived an improvement of over 60% in their eczema. These findings support the potential clinical utility of topical CBD in treating atopic dermatitis [123].

The study by Loewinger M. et al. (2022) evaluated the effectiveness of CBD and cannabidiolic acid (CBDA) in treating canine atopic dermatitis in 32 dogs. This prospective, randomised, double-blinded, placebo-controlled study administered 2 mg/kg of a CBD/CBDA mixture over 4 weeks. The treatment group showed significant reductions in pruritus but no improvement in skin lesions. Elevated alkaline phosphatase levels were noted in some dogs. The study concluded that CBD/CBDA reduced pruritus but did not affect skin lesions [126].

While preliminary findings from animal studies and small human trials are promising, further large-scale, randomised controlled trials are essential to confirm the efficacy and safety of CBD in managing atopic dermatitis [25,97,127].

### 7.5. Calciphylaxis

CBD has emerged as a promising therapeutic option for calciphylaxis—a rare, painful condition marked by skin necrosis due to vascular calcification, most commonly affecting patients with advanced kidney disease. As there is currently no definitive cure, treatment typically centres on symptom relief and wound management. Preliminary clinical observations suggest that topical CBD may support wound healing and alleviate pain, likely due to its anti-inflammatory, analgesic, and tissue-regenerative properties [128,129].

In a multicohort, open-label study conducted by Maida V. et al. (2020), 32 patients with non-uremic calciphylaxis received a topical formulation containing CBD (3.75 mg/mL) and a minimal dose of Δ^9^–THC (less than 1 mg per day), applied directly to wound beds and surrounding tissue. After one year of treatment, 90% of participants achieved complete wound closure. The study also included a single patient with uremic calciphylaxis, who presented with bilateral lower leg wounds. This patient demonstrated substantial granulation tissue formation—58% on the left leg and 78% on the right—alongside modest reductions in wound size (9% and 5%, respectively). No local or systemic adverse effects were reported, although the patient later died due to pre-existing cardiovascular disease [62,128].

Thus, the obtained evidence highlights the potential of topical CBD as a novel adjunctive therapy for managing calciphylaxis-related wounds. Further controlled studies are necessary to validate its efficacy and safety across broader patient populations.

### 7.6. Epidermolysis Bullosa

Emerging evidence suggests that cannabinoids may offer therapeutic benefits for various inflammatory skin disorders, including epidermolysis bullosa. Notably, sublingual administration of CBD has demonstrated effectiveness in alleviating epidermolysis bullosa-related pain [62].

In an observational study conducted by Chelliah M.P. et al. (2018), three cases of children with epidermolysis bullosa who independently began using topical CBD (1. CBD spray and tincture of CBD oil, 2. blend of emu oil and CBD oil, and 3. CBD oil and cream) were examined. One patient was successfully tapered off oral opioid analgesics entirely. All three individuals reported accelerated wound healing, reduced blister formation, and significant pain relief associated with the use of CBD [130].

In a 2019 case series, Schräder N.H.B. et al. (2019) reported on three individuals with epidermolysis bullosa who were treated with pharmaceutical-grade cannabinoid-based medicines containing both Δ^9^–THC and CBD, administered sublingually. All three patients experienced significant reductions in pain and pruritus (itching), alongside a decreased reliance on conventional analgesics [131].

Interesting results were provided by Schräder N.H.B. et al. (2021), obtained from an anonymous online survey. The study included 71 participants from five continents who reported using various cannabinoid preparations, most commonly via topical and oral routes, for treating epidermolysis bullosa. The most frequently used forms included oils or pastes, dried cannabis flower, and edibles such as infused or cooked foods. Participants reported substantial symptom relief, with pain and itching decreasing by an average of 3 points on a 10-point scale, and 95% of respondents noted overall symptom improvement. Additionally, 81% observed enhanced wound healing, and 79% reported a reduction in the use of traditional pain medications. The most commonly reported side effect was dry mouth. Topical application of CBD, in particular, was associated with significant subjective improvements in key symptoms of epidermolysis bullosa, including reduced pain and pruritus, as well as improved wound healing. The results suggest that topical CBD may provide localised therapeutic effects without systemic side effects, making it especially suitable for individuals with epidermolysis bullosa, whose skin is extremely fragile [131].

The study of Welponer T. et al. (2022) explored the use of purified oral CBD for managing pain in patients with severe recessive dystrophic epidermolysis bullosa, a rare and debilitating genetic skin disorder. Researchers administered high-purity (over 99.8%) oral CBD drops, free of Δ^9^–THC, as a palliative treatment to improve pain control and quality of life. The results indicated that CBD was well-tolerated and led to a significant reduction in pain intensity, a decreased need for opioid analgesics, and improved sleep and overall well-being in the patients. In conclusion, purified oral CBD may be a promising adjunctive therapy for pain management in severe recessive dystrophic epidermolysis bullosa cases [132].

A phase 2 clinical study (NCT05651607) is evaluating the efficacy of CBD on pruritus in children with hereditary epidermolysis bullosa (EBCBD) [133]. Further investigation through rigorously controlled clinical trials employing standardised cannabinoid formulations is necessary to accurately assess their safety and therapeutic efficacy.

### 7.7. Pruritus

Recent advances in dermatological research have highlighted cannabinoids as promising agents in the management of pruritus.

Pruritus is the most frequently reported skin-related symptom and is commonly associated with numerous dermatological conditions. It causes discomfort and can significantly affect a person’s quality of life. However, the exact biological mechanisms behind pruritus are still not fully understood [134].

Topical application and oral administration of cannabinoids have shown promise for treating itch, specifically in several diseases, including itch caused by skin inflammation, such as dermatitis, neurogenic itch associated with metabolic derangements, and chronic intractable itch in prurigo. Topical cannabinoids are increasingly favoured in dermatology for their safety profile and targeted application to affected skin regions. Products like creams, oils, and patches are now widely available. Because cannabinoids are lipophilic, they easily penetrate the skin, and to date, no systemic side effects have been reported from topical use. Importantly, many antipruritic benefits come from non-psychoactive cannabinoids like CBD and PEA, avoiding concerns about Δ^9^–THC-related effects [135].

CBD alleviates itch by interacting with the skin’s ECS, modulating receptors such as CB1, CB2, TRP channels, and serotonin receptors. The mechanism of the antipruritic effect of CBD is believed to involve anti-inflammatory, neuromodulatory, and barrier-restoring effects; however, further research is needed to fully elucidate the underlying pathways. CBD represents a promising candidate for topical antipruritic therapies, especially in conditions like atopic dermatitis, psoriasis, and eczema [97].

The preclinical study of Liu X. et al. (2023) highlights the crucial role of CB1R in managing inflammation and itch in psoriasis-like skin conditions. Activation of CB1R significantly reduced scratching and skin inflammation in a mouse model, while blocking it worsened symptoms. Researchers have found that CB1R is highly expressed in sensory neurons, particularly peptidergic ones, and its absence leads to an increased release of substance P, a neuropeptide that triggers inflammation and mast cell activation in the skin. Blocking substance P signalling reversed these effects, suggesting that CB1R helps control psoriatic inflammation and itch by regulating substance P in sensory neurons [136].

A clinical study evaluated the effectiveness of a cream containing structured physiological lipids and endogenous cannabinoids in treating uremic pruritus in 21 patients undergoing haemodialysis. For three weeks, patients applied the cream twice daily. Results showed a significant reduction in pruritus, with complete relief in 38.1% of participants and marked improvement in skin dryness (xerosis) in 81%. The treatment was well tolerated, and the findings suggest that the cannabinoids may have contributed to itch relief beyond just moisturising effects. However, further controlled studies are needed to confirm these results [137].

In a case study reported by Lou K. et al. (2022), a 60-year-old man with amyotrophic lateral sclerosis experienced persistent neuropathic pruritus that did not respond to standard treatments. Given the limitations of conventional therapies, a balanced oral cannabinoid from a licensed producer was chosen for its tolerability and evidence in managing neuropathic symptoms. The selected cannabinoid was a capsule containing 2.43 mg of Δ^9^–THC and 2.75 mg of CBD. The treatment led to a notable reduction in itch severity (from 7/10 to 3/10), with only temporary sedation. The case suggests that cannabinoids may be a safe and effective option for managing neuropathic pruritus, though further research is needed to confirm their efficacy and safety [138]. In cases of pruritus linked to chronic kidney disease, a cannabis-based topical cream significantly alleviated itching symptoms compared to a placebo treatment [139].

Clinical studies and some reports have shown that topical CBD formulations can significantly reduce the intensity of itching in patients with chronic pruritus [97]. For example, in a study, over 90% of patients reported a notable reduction in itch, with many reducing or discontinuing other anti-itch medications [140].

Thus, clinical evidence supports the effectiveness of CBD in topical and dietary applications as a promising, non-psychoactive treatment for pruritus, particularly in inflammatory skin conditions such as eczema and atopic dermatitis [25].

Overall, both preclinical and clinical evidence support the potential of cannabinoids, particularly non-psychoactive compounds such as CBD, as safe and effective treatments for various forms of pruritus. However, further research is essential to fully elucidate their mechanisms and optimise therapeutic use.

### 7.8. Psoriasis and Scalp Psoriasis

Plaque psoriasis is a persistent, immune-driven inflammatory skin condition that significantly impacts patients’ physical health, mental well-being, and social functioning. It is commonly linked with psoriatic arthritis and a range of other comorbidities, including cardiovascular disease, metabolic syndrome, and psychiatric disorders, all of which contribute to increased overall health risks and mortality. When psoriasis is limited to 3–5% or less of the body surface area, it is usually treated with topical agents, such as corticosteroids or non-steroidal formulations, and sometimes targeted phototherapy. However, if these approaches fail to produce adequate improvement, systemic therapies may be required to manage the disease more effectively [141].

CBD has emerged as a compound of significant interest in recent dermatological research, particularly for its potential therapeutic applications in the management of psoriasis and other skin disorders [25,62,142].

Several mechanisms of action of CBD in psoriasis are possible. CBD exerts therapeutic effects in psoriasis by reducing inflammation, modulating immune responses, normalising keratinocyte activity, and lowering oxidative stress through enhanced antioxidant defences [8,143]. CBD has been shown to inhibit key pro-inflammatory cytokines, such as TNF-α, IL-17, and IL-23, which are central to the pathogenesis of psoriasis. These cytokines are part of the TNF/IL-23/IL-17 axis, a well-established inflammatory pathway in psoriasis [144]. Evidence suggests that CBD exerts anti-inflammatory effects by inhibiting TNF-α-induced activation of the NF-κB transcription pathway, a key regulator of immune and inflammatory responses [145]. Keratinocytes play a crucial role in both initiating and maintaining psoriatic inflammation. CBD may help regulate their hyperproliferation and abnormal differentiation, which are hallmark features of the disease [146]. However, while not the primary focus of most psoriasis studies, CBD’s antioxidant properties, such as upregulating enzymes (e.g., SOD) and reducing ROS, are well-documented in broader cannabinoid research and contribute to its therapeutic potential [147].

Some relevant studies regarding the effect of CBD in psoriasis are presented below.

The study conducted by Kim M.-S. et al. (2024) explored the therapeutic potential of CBD in a mouse model of imiquimod-induced psoriasis. Mice were divided into five groups, including controls and those treated with clobetasol or two concentrations of CBD (0.01% and 0.1%). The application of CBD significantly reduced the severity of psoriasis, as measured by Psoriasis Area and Severity Index (PASI) scores, and improved histopathological features, including epidermal thickness and inflammation. Molecular analyses revealed that CBD suppressed the expression of key inflammatory cytokines (*IL-1b, IL-6, IL-12 b, IL-17a, IL-22,* and TNF) and inhibited the IL-23 receptor-mediated JAK2–STAT3 signalling pathway. Consequently, CBD may be an effective anti-psoriatic agent by modulating inflammatory and proliferative pathways [142].

A retrospective observational study evaluated the effects of a CBD-enriched topical ointment on 20 patients with common dermatological conditions, including psoriasis (n = 5). Participants applied the ointment to affected skin areas twice daily over three months. Clinical assessments—including skin hydration, TEWL, elasticity, and standardised indices such as the PASI, Scoring Atopic Dermatitis (SCORAD), and Acne Disability Index (ADI)—demonstrated significant improvements in both objective skin parameters and subjective symptom relief. Photographic documentation and clinical evaluations provided support for these results. Importantly, no adverse reactions were reported. The study concludes that topical CBD, free of THC, represents a safe and effective non-invasive therapeutic option for inflammatory skin disorders [106].

A clinical study evaluated the effects of a shampoo containing 0.075% broad-spectrum CBD in 50 individuals with mild to moderate scalp psoriasis or seborrheic dermatitis. Over 14 days, participants experienced significant reductions in inflammation, as measured by trichoscopic vascular patterns, and notable improvements in symptoms, including itching, burning, erythema, and scaling. Severity scores for these symptoms dropped markedly, and both tolerability and user satisfaction were rated as excellent. Thus, CBD-infused shampoo may offer a safe and effective short-term treatment for inflammatory scalp conditions [148].

Future research on CBD for psoriasis should focus on conducting large-scale, randomised controlled trials to confirm its efficacy and safety. Standardising formulations and dosages is also essential to ensure consistent outcomes. Additionally, exploring how CBD might work in combination with existing treatments could reveal synergistic benefits and improve therapeutic strategies. CBD shows promise as a complementary or alternative treatment for psoriasis due to its anti-inflammatory and immunomodulatory properties. However, further research is essential to confirm its long-term efficacy and safety through well-designed clinical trials.

### 7.9. Pyoderma Gangrenosum

Pyoderma gangrenosum is a rare, inflammatory skin condition characterised by deep, painful ulcers with well-defined, often violet or blue borders. It is considered a neutrophilic dermatosis and is frequently associated with systemic diseases, such as inflammatory bowel disease, arthritis, or hematologic disorders. Its exact cause is unknown, and diagnosis is clinical, usually requiring exclusion of other ulcerative skin conditions. Treatment often includes topical, oral, or intralesional corticosteroids, immunosuppressants, and biologics, such as TNF inhibitors [149].

To assess the effectiveness of topical medical cannabis in managing wound-related pain in three patients with pyoderma gangrenosum, a rare and painful skin condition, these patients were treated with topical cannabis oils containing both THC and CBD. Pain levels (0–10 scale) and opioid consumption (morphine sulphate equivalents, MSE) were measured before and after treatment. Statistical analysis involved paired t-tests. All three patients experienced rapid pain relief (within 3–5 min of application). The use of topical cannabis resulted in significant pain reduction in all cases. It showed both clinically and statistically significant relief in two instances and clinically meaningful relief in the third. It also demonstrated an opioid-sparing effect, which is essential amid the opioid crisis. The quick onset suggests a peripheral action mediated by cannabinoid receptors in the skin. CBD is thought to contribute to these effects through its anti-inflammatory and analgesic properties, interacting with the ECS present in the skin and wound tissues. The study supports further research into topical medical cannabis for wound pain management, particularly in conditions like pyoderma gangrenosum. Larger, controlled studies are recommended, as isolating the effects of CBD alone remains challenging [65].

### 7.10. Seborrheic Dermatitis

Seborrheic dermatitis is a prevalent papulosquamous skin condition that primarily affects infants and middle-aged individuals, with distinct presentations depending on age. It typically appears as salmon-coloured, follicle-centred papules and plaques covered with fine white scales and a greasy yellow crust, and can occur in various areas of the body, often affecting multiple sites simultaneously [150].

A clinical study evaluated the effects of a cream containing 3% cannabis seed extract on human cheek skin. Applied twice daily for 12 weeks, the cream significantly reduced both sebum production and erythema compared to a control. It was well tolerated, with no adverse skin reactions reported, suggesting its potential as a safe and effective treatment for oily skin conditions, such as acne and seborrhoea [151].

When used in a broad-spectrum shampoo formulation, CBD has demonstrated significant efficacy in treating seborrheic dermatitis by targeting key pathological mechanisms. CBD exhibits sebostatic properties, helping to regulate excessive sebum production, while also exerting potent anti-inflammatory and antioxidant effects. It modulates immune responses by suppressing pro-inflammatory cytokines and oxidative stress markers, and its lipophilic nature allows it to penetrate sebaceous glands effectively, delivering therapeutic benefits directly to the affected areas. In a clinical study involving 50 subjects with mild to moderate seborrheic dermatitis or scalp psoriasis, a shampoo containing 0.075% CBD resulted in marked improvements in inflammation, scaling, erythema, itching, and burning within two weeks. Trichoscopic evaluations showed significant reductions in arborising vessels and twisted capillaries, while patient-reported outcomes reflected high satisfaction and excellent tolerability. These results support CBD’s role as a promising topical agent for managing seborrheic dermatitis through its multifaceted biological actions [148].

Seborrheic dermatitis in Parkinson’s disease is often severe and resistant to topical treatments. The study of Weber I. et al. (2024) investigated whether oral CBD could reduce seborrheic dermatitis severity in individuals with Parkinson’s disease. Conducted as a randomised, double-blind, placebo-controlled trial, participants received either CBD or a placebo for 16 days. While the CBD group showed a trend toward improvement in seborrheic dermatitis severity compared to the placebo group, the results were not statistically significant. The study concluded that although oral CBD may have potential, the trial was underpowered to definitively assess its effect on seborrheic dermatitis, and further research is needed to confirm its therapeutic value in this context [152]. Cannabinoids, through their anti-inflammatory and neuromodulatory effects on the ECS, may offer therapeutic benefits for both conditions. The review by Rietcheck H.R. et al. (2021) examines the potential of oral cannabinoids in treating seborrheic dermatitis in patients with Parkinson’s disease, highlighting the need for further research on their safety and efficacy [153].

The discussed reports from the literature highlight the growing interest in cannabinoids as a promising therapeutic option for seborrheic dermatitis; however, further targeted studies are essential to confirm their efficacy, safety, and long-term benefits across diverse patient populations.

### 7.11. Skin Cancer

Cannabinoids are being explored as potential treatments for skin cancers, such as melanoma, squamous cell carcinoma, and Kaposi sarcoma. They have shown promise in preclinical studies by inhibiting tumour growth, spread, and blood vessel formation, while also promoting cancer cell death. These effects suggest that cannabinoids could be helpful for both the treatment and prevention of skin tumours [25,63,154,155].

Melanoma remains one of the deadliest forms of cancer, with limited success in preventing recurrence and overcoming resistance to current treatments. Cannabinoids have shown promising potential in the treatment of both melanoma and non-melanoma skin cancers by arresting cell cycles, inducing apoptosis, and employing other less-known mechanisms. Cannabinoid receptors CB1R and CB2R are present in melanoma cells, and their activation by cannabinoids can significantly reduce the viability of these cancer cells by inducing apoptosis. Notably, these effects appear to be selective for melanoma cells, sparing normal melanocytes despite their expression of CB1R [156,157,158].

The ECS, now recognised as a complex regulator of physiological balance across various body systems, including the skin, offers new therapeutic potential. The review by Bachari A. et al. (2024) explores the pathophysiology of melanoma, current pharmacological treatments, and their limitations, while emphasising the promise of cannabinoids as supportive agents. Cannabinoids may help restore immune balance and reduce inflammation in chronic skin conditions, presenting a novel approach to managing this highly immunogenic tumour [158].

Several melanoma cell lines were treated with varying concentrations of cannabinoids, including CBD, to evaluate their anti-cancer effects through proliferation and apoptosis assays. The study found that CBD, alone or in combination with THC, reduced cell viability in a dose-dependent manner, primarily through the activation of CB1, TRPV-1, and PPAR-α receptors. Blocking these receptors inhibited apoptosis. CBD triggered apoptosis by releasing mitochondrial cytochrome c, which subsequently activated caspases. In vivo experiments using NSG mice demonstrated that CBD significantly suppressed tumour growth, with efficacy comparable to that of the MEK inhibitor trametinib, without interfering with standard targeted therapies [159].

In animal models, the use of CB2R agonists and mixed CB1R/CB2R agonists, such as WIN-55,212-2, resulted in reduced tumour growth and metastasis, with treated tumours exhibiting increased apoptosis and decreased levels of Vascular Endothelial Growth Factor (VEGF), a key factor in tumour angiogenesis. Further studies in mice revealed that CBD administered intraperitoneally at a dose of 5 mg/kg twice weekly significantly reduced tumour size and improved survival rates compared to controls. While cisplatin treatment resulted in the most extended survival, CBD-treated mice exhibited better quality of life and mobility [160,161]. In addition, a combination of Δ^9^–THC and CBD was more effective than the standard chemotherapy drug temozolomide in reducing tumour growth and enhancing apoptosis in melanoma xenografts [162].

A study investigated the effects of two types of CBD oils on the migration and invasion of melanoma cells in vitro, aiming to explore their potential as alternative treatments for metastatic melanoma. Using scratch-wound and Boyden chamber assays, researchers found that both purified CBD and Charlotte’s Web CBD oil significantly reduced the metastatic potential of melanoma cells. Interestingly, purified CBD was more effective in reducing cell migration at higher concentrations, while both oils performed similarly in inhibiting cell invasion. The obtained results support the potential of CBD-based therapies in limiting the spread of melanoma [163].

A 64-year-old woman with a history of multiple squamous cell carcinomas presented with skin lesions on both of her dorsal hands. Biopsy results revealed lichen simplex chronicus on the left hand and squamous cell carcinoma on the right. Remarkably, both conditions resolved following the topical use of 20% CBD. The report also reviews skin-related side effects linked to cannabinoid use, as well as their potential therapeutic roles in treating inflammatory skin disorders and skin cancers [164].

In the context of Kaposi sarcoma, cannabinoids also showed therapeutic potential. CBD and the CB1R/CB2R agonist WIN-55,212-2 ((*R*)-[+]-[2,3-Dihydro-5-methyl-3-([(morpholinyl]methyl)pyrrolo(1,2,3-de)-1,4-benzoxazinyl]-[1-naphthalenyl]methanone)) both inhibited cell proliferation and induced apoptosis in Kaposi sarcoma cells [165]. CBD further suppressed the expression of viral proteins and VEGF [166]. However, low doses of THC had a counterproductive effect, enhancing viral infection and promoting tumour cell proliferation [167].

In summary, previous studies emphasise the importance of dosage and cannabinoid type in therapeutic applications, highlighting the need for further clinical trials to fully evaluate their efficacy and safety in treating skin cancers.

### 7.12. Wound Healing

CBD has gained attention for its therapeutic potential in wound care due to its anti-inflammatory, antioxidant, and analgesic properties. Wound healing is a complex biological process involving inflammation, tissue regeneration, and remodelling, and disruptions in these phases (especially in chronic wounds) can lead to delayed recovery. Recent studies suggest that CBD may modulate key immune responses and promote tissue repair by influencing cytokine activity, reducing oxidative stress, and enhancing the function of fibroblasts [60,128,168]. CBD is a promising compound in managing both acute and chronic wounds, particularly in conditions like diabetes, where healing is impaired.

However, the role of cannabinoid receptors in mediating wound healing responses is still under investigation, and it is unclear whether CBD acts directly through these receptors or via alternative pathways, such as TRPV-1 or PPAR-γ. A deeper understanding of these mechanisms is essential for optimising therapeutic strategies and identifying potential biomarkers of response.

Several in vitro studies focusing on CBD and wound healing have been published.

A study conducted by Monou P.K. et al. (2022) developed 3D-printed sodium alginate films incorporating nanoparticles of CBD and CBG, using Pluronic F127 as a carrier (a block copolymer formed of two hydrophilic ethylene oxide (EO) blocks and one hydrophobic propylene oxide (PO) block in an EOx–POy–EOx structure). In vitro wound healing assays showed that both CBD and CBG nanoparticles enhanced wound closure within the first 6 h of application. However, prolonged exposure beyond 6 h led to a reduction in healing efficacy. Cytotoxicity tests (MTT assay) revealed that a concentration of 0.1 mg/mL maintained good cell viability at 24 h, with CBG nanoparticles remaining non-toxic even at 48 h. Higher concentrations and longer exposure times significantly reduced cell viability. The obtained films have promising wound-healing potential when used at optimal concentrations and exposure durations [169].

Tran V.N. et al. (2023) developed a CBD nanoemulsion to address the compound’s limited stability and low bioavailability. They assessed its wound healing effectiveness using a laboratory model (in vitro) of the human cornea. The nanoemulsion maintained stable physicochemical properties for 90 days at room temperature and demonstrated significantly improved wound closure in scratch assays compared to both control and pure CBD. It also exhibited lower cytotoxicity and enhanced anti-inflammatory activity. Although neither the nanoemulsion nor pure CBD penetrated the cornea after four hours, the nanoemulsion retained 94% of its CBD content in the apical compartment, indicating reduced degradation compared to pure CBD. Overall, the nanoformulation enhanced the stability and biological activity of CBD, supporting its potential for wound healing applications [170].

A fibroin-based film dressing incorporating a CBD and 2-hydroxypropyl-β-cyclodextrin complex (CBD/HP-β-CD) was developed by Klinsang T. et al. (2023) and evaluated for its potential in wound healing. The CBD/HP-β-CD complex used in the film contains 81.5 ± 1.2% *w*/*w* CBD. The film exhibited strong mechanical properties and successfully encapsulated CBD, as confirmed by Fourier Transform Infrared Spectroscopy (FTIR) and X-ray Diffraction (XRD) analyses. In vitro studies using human dermal fibroblasts showed that the film enhanced cell proliferation, promoted fibroblast migration in scratch assays, and increased VEGF expression. Thus, the CBD/HP-β-CD fibroin film supports key cellular processes involved in wound repair, highlighting its promise as an effective wound-healing material [171].

CBD seem to enhance skin wound healing through multiple mechanisms, as demonstrated by animal studies. Research on animals indicates that applying cannabinoids might aid in the recovery of surgical and chronic wounds [60]. Recent studies based on animal models have been published.

The study conducted by Shah P. et al. (2024) investigated the effects of CBD on wound healing using both wild-type (C57BL/6) and diabetic (db/db) mice. In wild-type mice, CBD initially caused a delay in wound closure, though this difference diminished over time. In diabetic mice, CBD treatment led to a notable increase in connective tissue growth factor (CTGF) expression (from 18.6% to 38.8%), suggesting enhanced tissue regeneration. Additionally, CBD significantly reduced IL-33 levels in the wound by 70% in wild-type mice, indicating an anti-inflammatory effect. CBD could offer therapeutic benefits in impaired or chronic wounds, such as those associated with diabetes [168].

In a recent study by Lapmanee S. et al. (2025), a novel hydrogel scaffold incorporating cannabidiol-loaded lipid nanoparticles (CBD/LNPs) within a polyvinyl alcohol and sodium alginate matrix has demonstrated significant potential in promoting wound healing. The formulation enhances CBD stability and reduces cytotoxicity, while in vitro studies indicate that it accelerates fibroblast migration and mitigates oxidative stress. In vivo experiments on rats revealed that the CBD/LNP hydrogel significantly improved wound closure rates (by 35% by day 28) compared to controls, with histological analysis confirming enhanced epithelialisation, collagen deposition, dermal thickness, and reduced inflammation. The study highlights the scaffold’s dual anti-inflammatory and regenerative effects, positioning it as a promising therapeutic for chronic wound care [172].

While in vitro and animal studies provide promising results on the wound-healing potential of CBD, their translational value remains uncertain. Many of these studies utilise simplified models that do not fully replicate the complexity of human skin physiology or the multifactorial nature of chronic wounds. For instance, scratch assays and fibroblast migration tests, though useful, cannot account for the dynamic interplay between immune cells, extracellular matrix (ECM) components, and vascularisation processes in vivo. Moreover, the variability in CBD formulations, concentrations, and delivery systems across studies complicates direct comparisons and hinders the establishment of standardised therapeutic protocols.

However, there are limited human studies assessing the impact of CBD on wound healing, with most being case series and observational studies (Table 4) [60].

An early Phase 1 clinical study (https://clinicaltrials.gov/study/NCT05650697 (accessed on 27 June 2025)) aims to evaluate both the positive and negative effects of CBD on scar healing and appearance in patients who have undergone paramedian forehead flap reconstruction surgery [174].

A key challenge in using CBD for wound care is its poor stability and solubility. Although advanced delivery systems improve bioavailability, these technologies are still experimental, and their long-term safety in chronic wound settings remains unclear. In addition, most available studies are case reports or small-scale observational trials that lack control groups, randomisation, or blinding. Additionally, the absence of standardised dosing regimens and inconsistent reporting of CBD concentrations further weakens the reliability of the results. Without robust clinical trials, it is difficult to determine whether observed improvements are attributable to CBD itself or placebo effects, natural healing progression, or concurrent treatments.

Although more human studies are necessary, applying cannabinoids topically could be a promising treatment for postsurgical and chronic wounds.

## 8. The Role of Cannabidiol in Skin Rejuvenation and Ageing Prevention

CBD shows potential as an ingredient in anti-ageing skincare due to its properties, including antioxidant, anti-inflammatory, and moisturising [9,104,175]. Several preclinical studies are investigating the mechanisms by which CBD has anti-ageing effects on the skin.

Genetic deletion of the CB1 receptor in mice results in early cognitive decline and other age-related changes (hearing loss and reduced locomotor activity), which progress similarly in both genotypes. Additionally, early ageing-like changes were observed in the skin of CB1-deficient mice. Thus, the results suggest that CB1 receptor deficiency selectively accelerates cognitive and skin ageing without broadly hastening systemic ageing [176]. A deficiency of CB1 receptors in the skin may contribute to accelerated skin ageing by promoting oxidative stress through increased ROS production, weakening antioxidant defences, and enhancing pro-inflammatory responses. A marked reduction in CB1 receptor expression could play a key role in the early onset of ageing-like skin changes, particularly in conditions such as diabetes [177].

A study explored the molecular effects of CBD on primary human keratinocytes using RNA sequencing and mass spectrometry. CBD was found to regulate key pathways involved in skin and epidermal cell differentiation, and notably upregulated several Nrf2 target genes, especially HMOX1. CBD induced HMOX1 expression by promoting the degradation of the transcriptional repressor BACH1, independently of Nrf2. In vivo*,* topical CBD increased HMOX1 and keratins 16 and 17 (markers of skin repair and proliferation) in mouse skin [178].

CBD demonstrated significant anti-ageing and regenerative effects in both healthy and stress-induced premature senescent (SIPS) human skin fibroblasts. At concentrations between 0.5 µM and 2.0 µM, CBD enhanced cell proliferation and reduced cellular senescence, as indicated by decreased beta-galactosidase activity. It also promoted wound healing and modulated nuclear architecture. It influenced the expression of genes and proteins involved in cell cycle regulation and ECM production, including ELN, Cyclin D1, PCNA, and BID. Notably, in replicative senescent fibroblasts, CBD outperformed established anti-ageing agents, such as metformin, rapamycin, and triacetylresveratrol, in promoting wound repair, highlighting its potential as a bioactive compound for dermal rejuvenation and cosmetic applications [179].

Aqueous hemp leaf extract (CLES), rich in CBD (1.64% *w*/*w*) and phenolic compounds, demonstrated strong anti-photoaging properties in vitro. When applied to UVA-irradiated and aged fibroblasts, CLES significantly reduced oxidative stress, preserved type I procollagen levels, and suppressed the overproduction of MMP-1 and IL-6. Additionally, it lowered the percentage of senescence-associated β-galactosidase-positive cells from 18.8% to 11.9%, indicating a rejuvenating effect. Consequently, CLES is a promising cosmeceutical agent for preventing and treating photoaging [180].

A study conducted by Tassaneesuwan N. et al. (2025) found that pretreatment with CBD significantly reduced the number of senescent cells in both fibroblasts and keratinocytes exposed to oxidative stress induced by hydrogen peroxide (H_2_O_2_). Using senescence-associated β-galactosidase staining, researchers observed that CBD at concentrations of 1.25–2.5 µg/mL effectively decreased the percentage of senescent cells, comparable to the positive control (catechin). However, a higher concentration of CBD (5 µg/mL) did not reduce senescence and may have exacerbated oxidative stress, suggesting a dose-dependent effect. Thus, CBD has the potential to mitigate cellular ageing under oxidative conditions [181].

These studies demonstrate CBD’s ability to modulate key biomarkers, enhance cellular repair, and reduce oxidative stress, all of which are relevant to skin ageing. However, a critical limitation lies in the overreliance on preclinical data. While in vitro and animal models provide valuable possible mechanisms of action related to CBD, their findings may not fully translate to human physiology.

Although there is growing interest in CBD for its potential anti-ageing effects on the skin, clinical research in this area remains limited. Most existing evidence is derived from preclinical or in vitro studies, with only a small number of clinical trials registered or completed. Several relevant clinical studies are discussed below.

In a prospective, single-centre pilot study involving 10 participants aged 20 to 53 with facial skin imperfections, daily application of CR-Topical (a formulation containing 300 mg of water-soluble CBD, 0.2% retinol, proprietary botanicals, and stabilisers) over 42 days led to significant improvements in overall skin quality. Evaluations using the Global Ranking Scale (GRS) and 4-dimensional analysis showed the most notable improvements in visible pores, dehydration, surface roughness, and both static and dynamic wrinkles. Patient satisfaction was high, with 100% reporting positive outcomes and 90% willing to recommend the product to others. The formulation was well-tolerated, supporting its potential as an effective anti-ageing skincare treatment [182].

The combination of eicosapentaenoic acid (EPA) and CBD demonstrated enhanced anti-inflammatory and anti-ageing effects by significantly reducing the secretion of prostaglandin PGE2 and IL-8, key mediators of photoaging. Histological analysis indicated improved ECM remodelling post-UV exposure. At the same time, clinical assessments revealed notable reductions in crow’s feet and fine line wrinkles, as well as an 8.8% decrease in the subepidermal low-echogenic band (SLEB), a marker of skin ageing. Additionally, the formulation resulted in a significant reduction in the area and count of red spots, accompanied by notable improvements in skin hydration (31.2%) and elasticity (25.6%) after 56 days of application. The results were strongly supported by high participant satisfaction, highlighting the potent dermo-cosmetic and anti-ageing potential of the CBD-based formulation [183].

The anti-inflammatory and antioxidant properties of CBD may help reduce damage caused by UV-A exposure. McCormick E. et al. (2024) conducted a study on the use of topical nanoencapsulated CBD cream as a novel approach to counteract UV-A–induced damage to nuclear and mitochondrial DNA. Nineteen participants applied nano-CBD or vehicle cream to randomly assigned, blinded buttock sites twice daily for 14 days. The treated sites were then exposed to up to three times the minimal erythema dose of UV-A. After 24 h, punch biopsies were taken for histology, immunohistochemistry, and real-time polymerase chain reaction analysis. Applying nano-CBD cream topically decreased UV-A–induced formation of a common mutagenic nuclear DNA base lesion and safeguarded against mtDNA mutations linked to UV-A–induced skin ageing [80].

Although the few clinical studies cited are encouraging, they suffer from small sample sizes and short durations, which limit their statistical power and generalizability. Studies involving a small number of participants cannot robustly establish efficacy or safety across diverse populations. Another concern is the ambiguity concerning dose-response relationships (e.g., higher concentrations of CBD may exacerbate oxidative stress). Currently, comprehensive, large-scale clinical trials assessing long-term efficacy, safety, and mechanisms of action are still lacking. Comparative studies with established anti-ageing agents, exploration of optimal dosing strategies, and investigation into novel delivery systems are also warranted.

Furthermore, regulatory and safety considerations should be addressed, especially given the evolving legal landscape concerning CBD. In conclusion, the therapeutic potential of CBD for skin anti-ageing requires more rigorous clinical validation. A balanced approach that integrates mechanisms of action with clinical studies will be essential to establish CBD’s role in dermatological applications.

## 9. The Role of Cannabidiol in Oral Healthcare

CBD has recently attracted increasing interest in modern society and is being progressively investigated within the field of dentistry [184,185,186,187,188].

CBD exhibits a range of therapeutic properties, including anti-inflammatory, analgesic, antimicrobial, and osteoinductive effects, that make it a promising candidate for dental applications. CBD shows potential in endodontic therapy by promoting dental pulp regeneration, in periodontal treatment by reducing inflammation and bone loss, and in oral medicine for managing conditions like mucositis and bacterial infections [185,189].

In addition, CBD supports bone healing in oral surgery and may benefit patients with comorbidities such as cancer, arthritis, diabetes, and epilepsy. However, the field currently faces limitations, including the absence of standardised formulations, regulatory ambiguity, and a paucity of robust clinical trials [185].

In vitro and in vivo models demonstrate that CBD reduces key pro-inflammatory cytokines (TNF-α, IL-1β, and IL-6) and oxidative stress markers in oral tissues by modulating NF-κB signalling and enhancing antioxidant defences [187]. It also influences immune cell behaviour, promoting anti-inflammatory responses and tissue healing. Animal studies confirm its ability to reduce inflammation and preserve bone in periodontitis and peri-implantitis. Early clinical trials have reported benefits in pain relief, plaque reduction, and mucositis management, utilising delivery methods such as topical gels, mouthwashes, and microspheres. Broader clinical validation is needed [190,191].

Preclinical and in vitro studies demonstrate that CBD effectively disrupts pathogenic biofilms, suppresses inflammatory mediators, and promotes tissue regeneration—critical mechanisms in the prevention and management of oral infections such as dental caries, periodontitis, and candidiasis [191].

There are some possible administration routes, analysed by Hu Z. et al. (2024), for CBD in treating oral diseases. Oral administration is the most common and convenient method, but it is associated with low bioavailability due to CBD’s lipophilicity and gastrointestinal degradation. Sublingual delivery offers faster absorption and avoids first-pass metabolism, making it suitable for local oral conditions; however, saliva and swallowing can impact efficacy. Intravenous administration ensures rapid systemic effects but is invasive and impractical for routine dental care. Transdermal delivery offers sustained local drug release and is promising for treating chronic oral conditions, although systemic absorption is limited. Nasal administration enables rapid systemic delivery and potential targeting of the central nervous system; however, challenges persist in formulation. Other routes (pulmonary, ocular, rectal, and vaginal) may be used exceptionally or experimentally. Selecting the optimal route depends on the disease type, urgency, and treatment goals, highlighting the need for further research to optimise CBD delivery in dental applications [188].

A double-blind pilot study involving 40 patients evaluated the effects of a mouth rinse containing tea tree oil (TTO), spilanthol (a bioactive compound from *Acmella oleracea*, a member of the Asteraceae plant family), and CBD over 42 days. Participants used the rinse twice daily, and outcomes were measured using bleeding on probing, approximal plaque index (API), and oral microbiome assessments. All groups showed reductions in bleeding on probing and API, but the most significant improvements were observed in the test groups using the TTO + spilanthol + CBD combination. The combination demonstrated superior anti-inflammatory effects and better maintenance of oral microbiota balance compared to TTO alone, suggesting its potential as an effective adjunct in oral hygiene [192].

The clinical application of CBD in dentistry presents both challenges and promising opportunities. Key research gaps include understanding its pharmacodynamics, optimal dosing, and mechanisms of action in oral conditions, as well as evaluating patient acceptance and ensuring cytocompatibility. Standardised extraction methods and consistent testing protocols are urgently needed to ensure safety and efficacy. Although there is limited current research and patents, the presence of cannabinoid receptors in oral tissues and CBD’s broad pharmacological properties (anti-inflammatory, antioxidant, antibacterial, and neuroprotective) highlight its therapeutic potential. Future innovations may involve CBD-integrated tissue engineering, advanced drug delivery systems, and nanotechnology-based formulations to enhance bioavailability and precision. As clinical evidence grows, CBD could become a valuable, targeted tool in oral healthcare [184,188].

## 10. Toxicity and Side Effects of Topical Cannabidiol

Topical and transdermal CBD formulations have attracted growing research interest due to their prospective therapeutic roles in dermatological conditions. The existing evidence suggests that short-term use on human skin is generally well-tolerated, with no reports of allergic or irritating reactions [98].

This systematic review, conducted by Shevyrin V. et al. (2016), evaluated the efficacy and safety of skin-applied CBD formulations by analysing interventional human trials and plasma concentration data. Out of five hundred and forty-four screened articles, only eight met the inclusion criteria (three randomised controlled trials and five single-arm studies); eleven additional studies were identified but were inaccessible. The trials targeted dermatological conditions, pain, and behavioural symptoms, using CBD doses ranging from 50 to 250 mg or 0.075% to 1.0%, often in combination with other ingredients. However, high risk of bias, inconsistent methodologies, and poor reporting limited the reliability of findings. Although adverse events were rare and mild, their significance remains unclear without definitive efficacy data. The review also highlighted that achievable free CBD plasma levels in humans are low, potentially limiting therapeutic effects to high-affinity targets, while higher concentrations may pose safety risks. The authors concluded that current evidence is insufficient for generalising safety and efficacy across CBD products, emphasising the urgent need for well-designed, unbiased clinical trials that focus solely on CBD’s pharmacological effects [27].

There have been no recent systematic reviews on the side effects of topical or transdermal CBD; however, the limited available evidence suggests that it is safe and well-tolerated [9]. In a clinical study on topical CBD for digital ulcers in systemic sclerosis, 7 out of 25 patients (28%) reported mild side effects, including itching and perilesional redness; however, none discontinued treatment [62]. No severe adverse effects were observed, and physical examinations showed no changes in the perilesional skin. Other clinical studies have also reported no adverse effects associated with topical or transdermal CBD [193].

Overall, CBD appears to have a favourable safety profile, particularly in topical and transdermal forms. Further clinical trials with larger sample sizes, multiple doses, and various delivery systems are necessary to confirm its safety, particularly given the increasing global demand for CBD in cosmetic dermatology [9].

However, the current evidence base remains insufficient to draw definitive conclusions to establish the safety and efficacy of CBD in topical and transdermal applications.

## 11. Discussion

### 11.1. Market Trends and Consumer Demand

The cosmetics sector has rapidly evolved into one of the most dynamic and fast-expanding industries in recent years [194]. The skincare industry is experiencing a notable increase in consumer demand for natural, plant-based ingredients, with CBD emerging as a particularly prominent compound of interest. The CBD skin care market has emerged as a rapidly growing segment within the broader skincare and wellness industry. The global CBD skincare market was valued at approximately USD 964 million in 2022 (and 1.5 billion in 2023), and is projected to reach USD 5.4 billion by 2030 (8.9 billion by 2033), growing at a CAGR of 24.8% from 2022 to 2030 (a CAGR of 19.5% from 2024 to 2033) [195,196].

Consumers are increasingly drawn to multifunctional skincare products that offer anti-inflammatory, antioxidant, and anti-ageing benefits [194]. Multifunctional cosmetic ingredients are characterised by their capacity to provide a range of benefits, often combining protective, stabilising, and sensory-enhancing properties. These attributes enable them to address multiple skin concerns concurrently while maintaining the integrity and stability of the overall formulation [197]. CBD can be considered a multifunctional compound due to its numerous demonstrated biological effects (Section 5). In addition, biocosmetics have significant growth potential and emerging business opportunities, and there is an urgent need to transition from conventional fossil-based ingredients to natural, safe, and effective alternatives in cosmetic formulations [198].

As shown above, the CBD skincare market is projected to experience significant growth over the next decade, driven by increasing awareness of its therapeutic potential and a shift toward wellness-oriented beauty routines. The trend is further supported by the growing popularity of clean beauty, where transparency, sustainability, and minimalism are key drivers of purchasing [199].

The CBD skincare industry is expected to expand steadily, driven by increasing consumer interest in natural, multifunctional ingredients and wellness-focused beauty products. As clean beauty and sustainability become central to purchasing decisions, CBD’s therapeutic potential and versatility position it as a key player in the evolving cosmetics landscape.

### 11.2. Innovation in Formulations

Due to CBD’s lipophilic nature and poor water solubility, its effective topical and transdermal delivery remains a challenge, limiting its penetration beyond the skin surface. To overcome this, various advanced delivery systems have been developed to enhance the bioavailability and therapeutic efficacy of CBD (Section 6). These include chemical penetration enhancers, such as oleic acid, as well as innovative carriers, including hydrogels, liposomes, micelles, nanoemulsions, polymeric nanoparticles, and solid lipid nanoparticles. Physical enhancement techniques, such as microneedles and ultrasound, are also being explored. Among these, colloidal systems and nanoformulations have shown superior skin layer penetration in comparative studies.

Future CBD-based topical products are expected to benefit from advances in formulation science. Innovative delivery systems are being developed to enhance CBD’s skin penetration and stability. The new technologies aim to overcome CBD’s lipophilicity and poor water solubility, ensuring more effective delivery to target skin layers.

Additionally, synergistic formulations combining CBD with retinoids, hyaluronic acid, peptides, and botanical extracts are gaining traction due to their potential to enhance therapeutic outcomes in anti-ageing, acne, and barrier-repair products. While these combinations are present in commercial products, the scientific evidence on their synergistic mechanisms is still limited [97,182].

It is known that CBD is highly lipophilic, poorly water-soluble, and sensitive to light, oxygen, and heat, making it unstable in conventional formulations, as shown in Section 3.2. It degrades easily, especially in aqueous or alcohol-based systems, which limits shelf life and efficacy [50,51,52]. To overcome these challenges, researchers are utilising diverse technologies (Section 6), including hydrogels, liposomes, mycelles, and nanomicelles, as well as nanoemulsions and microemulsions, polymeric nanoparticles, SNEDDS, SLNs, and NLCs (Table 3, Section 6).

Innovative formulation technologies are crucial for unlocking the full potential of CBD in skincare. By overcoming challenges related to solubility, stability, and skin penetration, advanced delivery systems and synergistic combinations yield more effective and targeted CBD products. Continued research will be essential to validate these innovations and ensure their safe, science-backed application.

### 11.3. Regulatory Landscape and Standardisation

Previously, in Section 1.2, we discussed the regulations governing the use of cannabinoids in detail, with a particular focus on CBD. The regulatory framework for CBD in cosmetics remains fragmented globally. While some regions, such as the EU and the US, have established THC thresholds and ingredient sourcing guidelines, harmonised standards are still lacking [1,14]. Future developments are likely to focus on more precise definitions of permissible CBD sources, standardised testing for THC content, and stricter labelling requirements.

The lack of harmonised international standards for CBD has led to inconsistent product quality and safety. Regulatory bodies such as the EMA, FDA, and WHO have begun aligning on critical quality attributes (CQAs) for cannabinoid-based products, including cannabinoid content accuracy (typically 90–110% of label claim), limits on contaminants (e.g., heavy metals, pesticides, microbial load), and stability and shelf-life specifications [200]. The International Council for Harmonisation (ICH) has played a pivotal role in developing globally accepted guidelines for pharmaceutical quality, including Good Manufacturing Practice (GMP) and Good Clinical Practice (GCP), which are increasingly applied to cannabinoid products [201].

Regulatory convergence will be crucial to ensure product safety, efficacy, and consumer trust, particularly as the market matures and more clinical data become available [199]. Third-party laboratories are essential for verifying compliance with regulatory standards. These labs conduct cannabinoid profiling (including CBD, THC, and minor cannabinoids), contaminant screening, and stability testing under ICH conditions. Certification schemes such as ISO 17,025 [202] (testing labs), ISO 22,716 [203] (cosmetics GMP), and NSF/ANSI 173 (dietary supplements) are increasingly being adopted to ensure transparency and consumer trust [200].

The global CBD market continues to expand, and regulatory harmonisation is essential to ensure product quality, safety, and consumer confidence. Aligning international standards, enforcing accurate labelling, and adopting third-party certification will be key to supporting a transparent and trustworthy CBD industry.

### 11.4. Clinical Research and Evidence-Based Development

There is increasing consumer interest in topical cannabinoid-based products, which are widely marketed for cosmetic purposes under the guise of promoting health and well-being, and are regulated accordingly as cosmetic products. Formulations such as creams, ointments, gels, and patches often include cannabinoids such as CBD and THC, which are considered novel ingredients in the cosmetics industry. Preliminary studies suggest potential benefits of CBD in treating conditions such as acne, eczema, psoriasis, pruritus, and even skin ageing [9,61]. Most existing research is limited to in vitro, animal models, or small-scale human trials, often lacking rigorous design and placebo controls [61]. The classification of cannabis as a Schedule I substance in many jurisdictions has historically hindered large-scale clinical research. As a result, robust randomised controlled trials are urgently needed to validate CBD’s efficacy, safety, and mechanisms of action in dermatological applications [8,61].

However, concerns have been raised about potentially misleading claims regarding their dermatological benefits. There is an increasing need to investigate the clinical evidence supporting such claims by examining the current and emerging landscape of topical cannabis-based medicinal products [19]. There remains a need for robust, large-scale clinical trials to validate the efficacy and safety of CBD in dermatological applications.

Future research should focus on standardising dosages, identifying optimal delivery methods, and evaluating long-term effects. CBD’s interaction with the skin’s ECS opens promising avenues for future dermatological research. Key areas include [8,9,28,61,101,104,204]:

- Chronic inflammatory skin conditions (e.g., the treatment of atopic dermatitis, psoriasis, and contact dermatitis);
- Anti-ageing therapies (CBD has shown potential to reduce oxidative stress and inflammation, both of which contribute to skin ageing);
- Pigmentation disorders (ECS modulation may influence melanocyte activity, offering potential treatments for hyperpigmentation and vitiligo);
- Barrier repair (CBD may enhance skin hydration and barrier function by upregulating aquaporin-3 and modulating keratinocyte activity).

The rise of personalised skincare aligns well with CBD’s multifaceted pharmacological profile. Individual differences in skin type, ECS receptor expression, and inflammatory responses suggest that CBD-based treatments could be adapted to specific dermatological needs [8]:

- Sebum regulation in oily skin types may benefit from CBD’s anti-lipogenic effects [12];
- Sensitive or inflamed skin could respond well to CBD’s anti-pruritic and anti-inflammatory actions [135,136];
- Genetic profiling and microbiome analysis may further refine CBD-based regimens, enhancing efficacy and minimising adverse effects [205,206,207].

Consumer interest in natural and holistic skincare is growing. Integrating CBD into personalised dermatological care could revolutionise treatment paradigms, provided that future research substantiates its benefits. Although early studies suggest promising dermatological benefits of CBD, robust clinical evidence remains limited. Advancing research through well-designed trials and personalised approaches will be essential to validate CBD’s efficacy and safety in skincare, ensuring its integration into evidence-based dermatological practice.

### 11.5. Sustainability and Ethical Considerations

Sustainability will play a pivotal role in shaping the future of CBD skincare. Eco-friendly sourcing of hemp and CBD involves sustainable agricultural practices that minimise environmental impact while ensuring product quality. Hemp is inherently sustainable due to its low need for pesticides, rapid growth cycle, and ability to improve soil health through phytoremediation. Organic farming, crop rotation, and water-efficient irrigation are crucial in reducing the ecological footprint of hemp cultivation [208].

Furthermore, the development of high-CBD, low- Δ^9^–THC hemp varieties supports both therapeutic applications and environmental goals by enabling broader cultivation under legal frameworks. Aftab N. et al. (2024) investigated the genetic diversity of 54 *Cannabis sativa* accessions from tropical and subtropical regions of India. The researchers identified specific accessions, such as CIM-CS-64, that exhibited high CBD content with extremely low Δ^9^–THC levels (as low as 0.01%). The accessions were highlighted as promising candidates for breeding programs aimed at producing medicinal hemp varieties with high CBD and compliant Δ^9^–THC levels for legal cultivation [209]. The practices are complemented by transparent supply chains and eco-conscious processing methods, which are increasingly adopted by responsible CBD producers [208].

Singh S. et al. (2025) provide a comprehensive analysis of biodegradable packaging films, focusing on their sources (e.g., polysaccharides, proteins, lipids), additives (such as nanocomposites and bioactive compounds), and preparation methods, including extrusion and solution casting. The materials are increasingly used in food and pharmaceutical packaging due to their ability to retain moisture, provide oxygen barrier properties, and exhibit environmental degradability [210]. Nizamuddin S. and Chen C. (2024) explore the chemical nature and degradation pathways of biobased, biodegradable, and compostable plastics. It was emphasised that clear definitions and international standards are crucial for ensuring proper labelling and disposal. Some materials, including polylactide (PLA) and starch-based polymers, are increasingly used in packaging to reduce the environmental footprint of products [211].

Kaur et al. (2024) discuss the role of green chemistry in producing biodegradable plastics, emphasising principles such as waste prevention, atom economy, and the use of renewable feedstocks. Additionally, it highlights how green synthesis methods and safer solvents are applied to develop bioplastics, such as PLA and polyhydroxyalkanoates (PHA), which are suitable for sustainable packaging in various industries [212].

The integration of biodegradable packaging and green chemistry principles is particularly relevant to the CBD industry, which is increasingly aligning with consumer demand for environmentally responsible products. As CBD oils, tinctures, and topicals often require packaging that ensures product stability and safety, the adoption of bioplastics such as PLA and PHA offers a sustainable alternative to conventional petroleum-based plastics. These materials not only reduce the environmental footprint of packaging but also align with the natural and wellness-oriented branding of CBD products. Moreover, the application of green chemistry in CBD formulations, such as the use of safer solvents and renewable feedstocks, enhances product safety and minimises ecological impact throughout the production lifecycle. This holistic approach supports a circular economy model, reinforcing the CBD sector’s commitment to sustainability and regulatory compliance.

Ethical marketing and transparency in labelling are crucial components in the industry, where consumer trust and regulatory compliance are paramount. Ethical branding involves a deliberate and continuous effort to align business practices with moral principles, including honesty, accountability, and social responsibility [213]. In the context of CBD, it includes communicating product contents, sourcing practices, and health claims without exaggeration or misleading information. Transparency in labelling is essential not only for consumer safety but also for fostering long-term brand loyalty. Ethical marketing goes beyond legal compliance to address broader societal expectations and stakeholder concerns. Moreover, ethical marketing must be culturally sensitive and avoid greenwashing or exploitative tactics, especially in wellness sectors like CBD, where consumers are particularly attuned to authenticity and sustainability [214,215].

For example, the 2018 USA Farm Bill legalised hemp-derived products with less than 0.3% Δ^9^–THC [15], creating a new market for CBD and Δ^8^–THC. However, the FDA has not yet effectively regulated these products, leading to concerns about misinformation and safety. The proposed solution includes stricter regulation, removing Δ^8^–THC from legal status, and treating CBD as a dietary supplement to ensure consumer protection and product transparency. The proposal recommends that the FDA establish more effective strategies to address misinformation about CBD products and increase enforcement by issuing additional warning letters to eliminate deceptive items from the market. This approach would enable proper FDA oversight of the CBD industry, ensuring consumers receive accurate information and can make safer purchasing decisions [216].

Ethical considerations in the use and marketing of CBD products revolve around transparency, patient safety, and regulatory compliance. Many CBD products are inaccurately labelled, often misrepresenting their CBD and Δ^9^–THC content, and may contain harmful impurities such as pesticides and heavy metals. The mislabelling poses risks not only to consumers but also to health systems that may inadvertently violate federal or state laws. Ethical practice demands that health care providers, especially pharmacists, be well-informed and cautious when advising patients, ensuring that decisions are based on evidence rather than marketing claims. Furthermore, institutions must develop clear policies to manage patient-owned CBD products, striking a balance between patient autonomy and legal and safety responsibilities [217].

The future of CBD skincare lies in striking a balance between innovation and responsibility. Sustainable sourcing, biodegradable packaging, and ethical marketing are no longer optional because they are essential. By embracing transparency, environmental stewardship, and consumer safety, the CBD industry can establish lasting trust and make meaningful contributions to a more sustainable and ethical wellness market.

### 11.6. Challenges and Opportunities

The integration of CBD into dermocosmetic formulations presents both significant challenges and promising opportunities. One of the primary technical challenges lies in CBD’s physicochemical properties, including its lipophilicity, poor water solubility, and instability under exposure to light, heat, and oxygen [6,48,49,50]. These characteristics complicate formulation development, limiting CBD’s bioavailability and therapeutic efficacy in topical applications. Overcoming these barriers requires the continued advancement of innovative delivery systems, such as nanoemulsions, micelles, liposomes, and solid lipid nanoparticles (Table 3, Section 6), which have shown promise in enhancing skin penetration and protecting CBD from degradation [109]. HPLC and GC-MS analyses have been used to identify and quantify major cannabinoids. Thus, comprehensive profiling of all CBD derivatives in plant extracts is still an emerging area of research. Future studies should aim to characterise the full spectrum of CBD-related compounds in cosmetic-grade extracts to better understand their therapeutic contributions [6,24].

Another major challenge is the widespread misinformation and exaggerated marketing claims surrounding CBD-based skincare products. Many commercial formulations lack standardisation, and product labelling often fails to accurately reflect cannabinoid content or purity, leading to diminished consumer trust and increased regulatory concerns. Addressing this issue necessitates greater transparency, third-party testing, and adherence to emerging international standards (Section 11.3). Educating consumers and healthcare professionals about the science behind CBD’s dermatological effects is crucial for dispelling myths and promoting evidence-based use.

However, the CBD skincare sector offers numerous opportunities. Niche markets, such as post-procedure recovery, sensitive skin, and men’s grooming, are underexplored and present opportunities for targeted product development. CBD’s multifunctional properties, including anti-inflammatory, antioxidant, analgesic, and moisturising effects (Section 7), make it an ideal candidate for addressing complex skin concerns in these specialised segments. Furthermore, the convergence of CBD with emerging technologies, such as artificial intelligence (AI)-driven skin diagnostics and personalised skincare regimens, opens new frontiers for precision dermatology [218,219]. As scientific understanding deepens and regulatory frameworks mature, CBD is poised to transition from a trend-driven ingredient to a validated therapeutic agent in dermatological and cosmetic science [3,9,181].

Additionally, misinformation and exaggerated marketing claims pose risks to consumer confidence [216,217,220]. The rapid rise in CBD popularity, fuelled by its portrayal as a “cure-all”, has led to the proliferation of over 1000 products. Limited research exists on how consumers choose CBD for medical use. A thematic analysis of 164 GoFundMe campaigns revealed that individuals often turn to CBD based on self-directed research, recommendations from trusted healthcare providers, or anecdotal experiences within their social networks. Additionally, an analysis of 2165 CBD products on Canadian websites showed that marketing frequently frames CBD as a treatment or cure, a natural health product, or a targeted solution for specific outcomes. The findings highlight the necessity of systematic regulation and auditing of CBD product claims in order to safeguard consumers and support evidence-based application [221].

However, these challenges also present opportunities for innovation. Companies that invest in research, transparency, and education will be well-positioned to lead the next wave of CBD skincare.

Niche markets, such as post-procedure recovery, sensitive skin, and men’s grooming, offer untapped potential for targeted CBD formulations. In post-procedure care, CBD’s anti-inflammatory and soothing properties can support skin regeneration and reduce discomfort following dermatological treatments, such as laser therapy or chemical peels. For individuals with sensitive skin, CBD’s ability to modulate inflammatory pathways and reinforce the skin barrier makes it a promising alternative to harsher actives. Additionally, the men’s grooming segment, often underserved in skincare innovation, can benefit from CBD’s calming and anti-irritant effects, particularly in products designed for shaving, beard care, or post-shave recovery [9,60,63,222,223]. Applications of CBD align with growing consumer demand for personalised, functional skincare solutions and present a strategic opportunity for brands to differentiate themselves in a competitive market.

CBD’s integration into skincare faces formulation and regulatory challenges. Thus, it also presents unique opportunities for innovation. With its multifunctional properties and growing consumer interest, CBD is well-positioned to address unmet needs in niche markets such as post-procedure care, sensitive skin, and men’s grooming. As scientific validation and regulatory clarity improve, CBD is likely to evolve into a trusted therapeutic agent in dermatological and cosmetic applications.

## 12. Conclusions

Several scientific advancements concerning the dermatological and cosmetic applications of CBD were synthesised in this comprehensive review. Specifically, CBD exhibits different biological effects on the skin, mediated through its interaction with the ECS and multiple molecular targets. Its anti-inflammatory, antioxidant, antibacterial, analgesic, and antiproliferative properties contribute to its therapeutic potential in managing inflammatory and immune-mediated skin disorders, as well as in promoting skin rejuvenation, hydration, and protection against oxidative and environmental damage.

Although CBD exhibits promising therapeutic potential, the use of CBD in dermatology and cosmetics remains constrained by several factors, including suboptimal physicochemical characteristics, limited transdermal permeability, the absence of standardised formulations, and variability in regulatory frameworks. Additionally, safety concerns and the need for more robust clinical evidence persist. Future research should focus on optimising delivery systems, clarifying mechanisms of action, and conducting large-scale clinical trials to confirm efficacy and long-term safety. With continued innovation and scientific validation, CBD may become a valuable and well-integrated active ingredient in dermatological and cosmetic therapies, as well as in emerging applications within oral health care.

## Figures and Tables

**Table 1:** A classification system of cannabinoids [27].

Phytocannabinoids	Endocannabinoids	Synthetic Cannabinoids
Tricyclic terpenoids	AEA pathway	Phytocannabinoid-similar (tricyclics, bicyclics, other)
Bicyclic terpenoids	2-AG pathway	Endocannabinoid-related (eicosanoid-similar, endocannabinoid modulators)
Other	Other eicosanoids	Indole-similar (indoles, indazoles, benzimidazoles, other azaindoles)
		Indenes (naphthylmethylindenes)
		Pyrrole-similar (pyrroles, pyrrazoles)
		Carbazole-similar
		Miscellaneous

**Table 2:** Physicochemical properties of cannabidiol (CBD) (CAS Number: 13956-29-1); Ref. = references.

No.	Properties	Data	Ref.
1	Molecular weight	314.46 g/mol	[22,53]
2	Water solubility	0.0126 mg/mL (ALOGPS)	[48]
3	DMSO solubility	To 75 mM	[53]
4	Ethanol solubility	To 75 mM	[53]
5	p*K*_a_ (Strongest Acidic)	9.13 (Chemaxon)	[48]
6	p*K*_a_ (Strongest Basic)	−5.7 (Chemaxon)	[48]
7	log*P*	6.6; 6.33 (Chemaxon); 6.1	[48,49,53]
8	ALogP	5.85	[54]
9	log*S*	−4.4 (Chemaxon)	[48]
10	Log D 7.4	6.53	[55]
11	CXlog*D*	6.32	[54]
12	Melting point	62–63 °C; 66–67 °C	[42,53]
13	Density	945 and 980 kg/m^3^	[56]
14	Form	Powder	[53]
15	Colour	White	[53]
16	Storage temperature	2–8 °C	[53]

**Table 3:** Advanced CBD skin delivery systems.

System Type	Mechanism	Advantages	Limitations	Ref.
Hydrogels	Hydrophilic networks retain CBD for prolonged contact	Moisturising and prolonged skin contact	Limited drug loading capacity	[97]
Liposomes	Encapsulate CBD within phospholipid bilayers, facilitating fusion with skin lipids	Enhance skin penetration, biocompatible, and protect CBD from degradation	Stability issues, potential for oxidation	[109]
Micelles and nanomicelles	Self-assemble from amphiphilic molecules into core-shell structures that solubilise CBD in the hydrophobic core	Improve solubility, stability, and targeted delivery of poorly water-soluble CBD	Sensitive to dilution and environmental changes; potential for premature release	[110]
Microneedles	Create microchannels in the skin to bypass the stratum corneum and deliver CBD directly	Bypass the stratum corneum, enhance delivery to deeper layers	Invasive, may cause discomfort or irritation	[9]
Nanoemulsions and microemulsions	Enhance solubility and penetration; controlled release	Improved stability and spreadability	May require surfactants; stability issues	[97,110,111]
Polymeric nanoparticles	Encapsulate CBD in biodegradable polymers	Sustained release and protection from degradation	Polymer toxicity and complex synthesis	[97]
Self-Nanoemulsifying Drug Delivery Systems (SNEDDS)	Form nanoemulsions on contact with moisture	Enhanced solubility and absorption	Formulation sensitivity to oils and surfactants	[110]
Solid Lipid Nanoparticles (SLNs) and Nanostructured Lipid Carriers (NLCs)	Use lipid matrices for controlled release and stability	Higher drug loading and stability (NLCs)	SLNs have limited drug loading	[112]

**Table 4:** Studies in which CBD was administered to humans (Ref. = reference).

No.	Year	Topical Forms	Dose of CBD	Results	Ref.
1	2018	Topical tincture of CBD oil, CBD oil and cream	Unreported data	Patients with epidermolysis bullosa experienced symptom improvement (faster wound healing, less blistering, and amelioration of pain)	[130]
2	2019	Topical CBD ointment	Unreported data	The skin parameters, symptoms, and PASI index score of 20 patients with common skin disorders (5 with psoriasis, 5 with atopic dermatitis, and 10 with resulting outcome scars) significantly improved	[106]
3	2020	Topical mixtures of cannabinoids, terpenes, and flavonoids, applied topically to the wound beds and peri-wound tissues	375 mg/mL	Two patients with painful and non-healing leg ulcers, of greater than 6 months duration, experienced rapid wound closure and relief of wound-related pain	[129]
4	2021	Mixtures of cannabinoids, terpenes and flavonoids, applied topically to the wound beds and peri-wound tissues	3.8 mg/mL	14 patients with 16 chronic and non-healing leg ulcers experienced rapid wound closure in previously non-healing wounds	[173]
5	2021	CBD CBD + Δ^9^–THC	Unreported data	Patients with epidermolysis bullosa reported wound healing	[140]

## Data Availability

Not applicable.

## Abbreviations

The following abbreviations are used in this manuscript:

AEA	Anandamide
ALOGPS	Associative Logarithm of Partition Coefficient and Solubility
API	Approximal plaque index
ARTG	Australian Register of Therapeutic Goods
AQP3	Aquaporin-3
BACH1	BTB domain and CNC Homology 1
CBD	Cannabidiol
CB1	Cannabinoid Receptor Type 1
CB2	Cannabinoid Receptor Type 2
CBDA	Cannabidiolic acid
CBG	Cannabigerol
CBC	Cannabichromene
CBN	Cannabinol
DMSO	Dimethyl sulfoxide
DWC	Dermal water content
ECS	Endocannabinoid system
EMA	European Medicines Agency
EPA	Eicosapentaenoic acid
EU	European Union
FAAH	Fatty acid amide hydrolase
FDA	Food and Drug Administration
GCP	Good Clinical Practice
GMP	Good Manufacturing Practice
HMOX1	Heme oxygenase 1
IL	Interleukin
MAPK	Mitogen-Activated Protein Kinase
MITF	Microphthalmia-Associated Transcription Factor
MRSA	Methicillin-Resistant *Staphylococcus aureus*
NLCs	Nanostructured Lipid Carriers
Nrf2	Nuclear Factor Erythroid 2–Related Factor 2
PASI	Psoriasis Area and Severity Index
PEA	N-Palmitoylethanolamine
PPAR	Peroxisome Proliferator-Activated Receptor
ROS	Reactive Oxygen Species
SAS	Special Access Scheme
SLEB	Subepidermal Low-Echogenic Band
SNEDDS	Self-Nanoemulsifying Drug Delivery Systems
SOD	Superoxide Dismutase
TEWL	Transepidermal water loss
THC	Tetrahydrocannabinol
THCA	Tetrahydrocannabinolic acid
THCV	Tetrahydrocannabivarin
TRP	Transient receptor potential
TRPA	Transient receptor potential ankyrin
TRPV	Transient receptor potential vanilloid
UK	United Kingdom
USA	United States of America
VEGF	Vascular Endothelial Growth Factor
VRSA	Vancomycin-resistant *Staphylococcus aureus*
